# Metallosalen modified carbon nitride a versatile and reusable catalyst for environmentally friendly aldehyde oxidation

**DOI:** 10.1038/s41598-024-58946-3

**Published:** 2024-04-11

**Authors:** Reza Eskandari Sedighi, Mahdi Behzad, Najmedin Azizi

**Affiliations:** 1 Faculty of Chemistry, Semnan University, Semnan, Iran; 2 Chemistry and Chemical Engineering Research Center of Iran, P.O. Box 14335-186, Tehran, Iran

**Keywords:** Carbon nitride, Metallosalen, Aldehyde, Green chemistry, Oxidation, Hydrogen peroxide, Chemistry, Catalysis, Green chemistry, Organic chemistry

## Abstract

The development of environmentally friendly catalysts for organic transformations is of great importance in the field of green chemistry. Aldehyde oxidation reactions play a crucial role in various industrial processes, including the synthesis of pharmaceuticals, agrochemicals, and fine chemicals. This paper presents the synthesis and evaluation of a new metallosalen carbon nitride catalyst named Co(salen)@g-C_3_N_4_. The catalyst was prepared by doping salicylaldehyde onto carbon nitride, and subsequently, incorporating cobalt through Schiff base chemistry. The Co(salen)@g-C_3_N_4_ catalyst was characterized using various spectroscopic techniques including Scanning Electron Microscopy (SEM), X-ray Diffraction (XRD), Infrared Spectroscopy (IR), and Thermogravimetric Analysis (TGA). Furthermore, after modification with salicylaldehyde, the carbon nitride component of the catalyst exhibited remarkable yields (74–98%) in oxidizing various aldehyde derivatives (20 examples) to benzoic acid. This oxidation reaction was carried out under mild conditions and resulted in short reaction times (120–300 min). Importantly, the catalyst demonstrated recyclability, as it could be reused for five consecutive runs without any loss of activity. The reusable nature of the catalyst, coupled with its excellent yields in oxidation reactions, makes it a promising and sustainable option for future applications.

## Introduction

Selective oxidation of aldehydes to acids is one of the most important reactions in the chemical industry^1^. Among these reactions, the oxidation of benzyl alcohol to benzaldehyde and its conversion into valuable products in the fragrance, dye, pharmaceutical, and chemical industries make it a significant chemical process^2–4^. In recent decades, the development of processes utilizing safe and economically feasible oxidants has attracted considerable attention from researchers^5–8^. The oxidation of aldehydes using molecular oxygen (O_2_) as the oxidizing agent has garnered significant research interest due to its environmentally friendly nature and low cost^9–12^. Furthermore, the selection of a desirable catalyst and the avoidance of hazardous solvents are also crucial aspects of these processes^13–17^. Heterogeneous catalysts present notable advantages over homogeneous catalysts, due to their easy separation from the reaction medium and the possibility of catalyst reuse^18–20^.

In recent decades, Schiff bases have played a key role as chelating ligands in coordination chemistry of transition metals and main group metals^21^. These ligands can easily form stable complexes with a variety of metal ions^22,23^. Transition metal complexes with oxygen- and nitrogen-donor Schiff base ligands have special significance due to their ability to adopt diverse configurations, structural flexibility, and sensitivity to molecular environments^24,25^. Metal complexes formed by Schiff base ligands that have both hard donor atoms such as oxygen and nitrogen and soft donor atoms such as sulfur often exhibit interesting physical and chemical properties^26,27^. Schiff bases have diverse applications in various fields such as industry, medicine, and chemical synthesis^28,29^. Their properties include antimicrobial and antibacterial effects, anticancer, antioxidant, and catalytic properties, as well as applications in organic synthesis, photochromism^30–32^, and other applications, which have attracted the attention of researchers to prepare various types of Schiff bases through bifunctional reactions^33–35^.

Graphitic carbon nitride (g-C_3_N_4_) is a carbon-based material that has gained attention in various research fields^36–40^, including catalysis, energy storage, optoelectronics, and environmental applications^41–44^. It offers high chemical stability and a tunable bandgap, making it suitable for optoelectronics and photocatalysis^45–48^. In photocatalysis, it has shown excellent performance in water splitting, pollutant degradation, and carbon dioxide reduction^49,50^. Carbon nitride also has interesting electronic properties as a semiconductor, which can be modified for enhanced charge transport in electronic devices like sensors and transistors^51–53^.

Our continuous interest in carbon nitride chemistry and our ongoing research aims to explore new synthetic strategies, optimize catalytic performance, and expand the scope of carbon nitride-based catalysts in various organic transformations^54–56^. Considering the presence of the nitrogen functional group on carbon nitride, this inexpensive platform can be used as a support and Schiff base agent. In this article, the advantage of carbon nitride was utilized for the preparation of Schiff base complexes involving copper, cobalt, and manganese. Carbon nitride was used as a support material as well as the Schiff base agent. The nitrogen atoms in carbon nitride was reacted with salicylaldehyde to provide a stable environment for the formation of Schiff base complexes. This approach allows for the immobilization of metal ions on carbon nitride, leading to the synthesis of supported Schiff base catalysts.

## Experimental

### Preparation of g-C_3_N_4_

The synthesis of bulk g-C_3_N_4_ involved a direct heating method using melamine in air, following the procedure outlined in the previous paper^23^. 20 g (158 mmol) of melamine powder was placed into a covered 50 mL alumina crucible and was subjected to gradual heating in a muffle furnace. The temperature was increased at a rate of 5 °C per minute until reaching a final temperature of 550 °C and maintained at 550 °C for 4 h. After the reaction time, the crucible was allowed to cool down naturally to room temperature. A light-yellow powder, which corresponds to the synthesized bulk g-C_3_N_4_, was obtained and collected from the crucible. The g-C_3_N_4_ nanosheets was prepared through thermal exfoliation. The 5 g of bulk g-C_3_N_4_ was placed into an uncovered crucible. The crucible with the bulk g-C_3_N_4_ was subjected to heat treatment in a furnace at a temperature of 550 °C for 3 h. After the heat treatment, a white powder, consisting of g-C_3_N_4_ nanosheets, was obtained.

### Preparation of Co(salen)@g-C_3_N_4_

**Figure 1:**
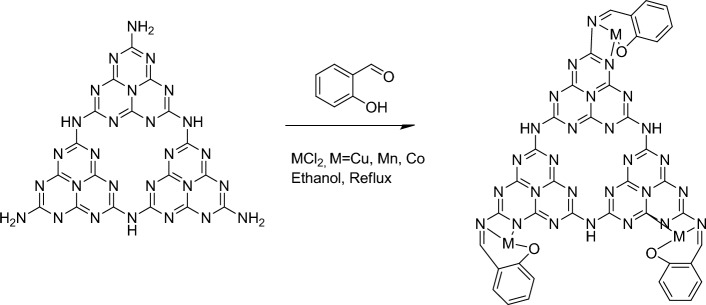
Preparation of the Co(salen)@g-C_3_N_4_ catalysts.

1 g of g-C_3_N_4_ was added to a dry ethanol solution (50 mL) and the mixture was an ultrasound with ultrasonic probe for 10 min to ensure proper dispersion of g-C_3_N_4_ in ethanol. To the g-C_3_N_4_ suspension, 0.5 g (4 mmol) of salicylaldehyde was added. The resulting suspension was refluxed for 24 h with constant stirring. The solution was allowed to cool to room temperature. It was then washed twice with 15 mL portions of ethanol. 1.0 g of salen@g-C_3_N_4_ was dispersed in 50 mL of dry ethanol and 0.5 g (4 mmol) of cobalt(II) chloride was added. The resulting solution was stirred at reflux for 8 h. After the refluxing period, the solid product was washed twice with 15 mL portions of ethanol and dried (Fig. 1).

### Experimental procedure

In a test tube equipped with a magnetic stirring bar and septum was charged with Co(salen)@g-C_3_N_4_ (30 mg), aldehydes (1 mmol), and H_2_O_2_ (3 mmol). The mixture was heated at 60 °C with stirring until the reaction was complete, followed by cooling to room temperature. Water (10 mL) was added to quench the reaction, and the resulting mixture was then extracted with ethyl acetate (10 mL). The organic layer was dried using MgSO_4_, and the solvent was evaporated under vacuum. In most cases, the reaction products were obtained in high purity and did not require additional purification methods. ^1^H and ^13^C NMR analysis and comparison of the melting points with literature values confirmed the identity of the compounds. In a few cases, crude products were further purified using silica column chromatography with ethyl acetate and petroleum ether as eluents.

## Results and discussion

**Figure 2:**
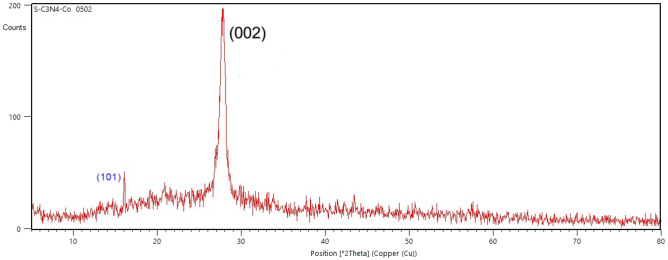
PXRD pattern of Co(salen)@g-C_3_N_4_.

The characterization of Co(salen)@g-C_3_N_4_ crystalline structures was carried out using powder PXRD (Fig. 2). The PXRD patterns ^12^ of Co(salen)@g-C_3_N_4_ showed two major peaks of diffraction at approximately 27.7° and 13.1°, as depicted in Fig. 1. The peak at around 27.7° was identified as the (002) plane, which arises from the interplanar stacking of aromatic systems.

A slight decrease in the peak intensity was observed after the incorporation of the Co complex compared to that of unmodified carbon nitride. However, it was observed that the functionalization of g-C_3_N_4_ with the Co complex did not alter the crystalline structure as with pure g-C_3_N_4_ (Figure S1 in Supporting Information).

**Figure 3:**
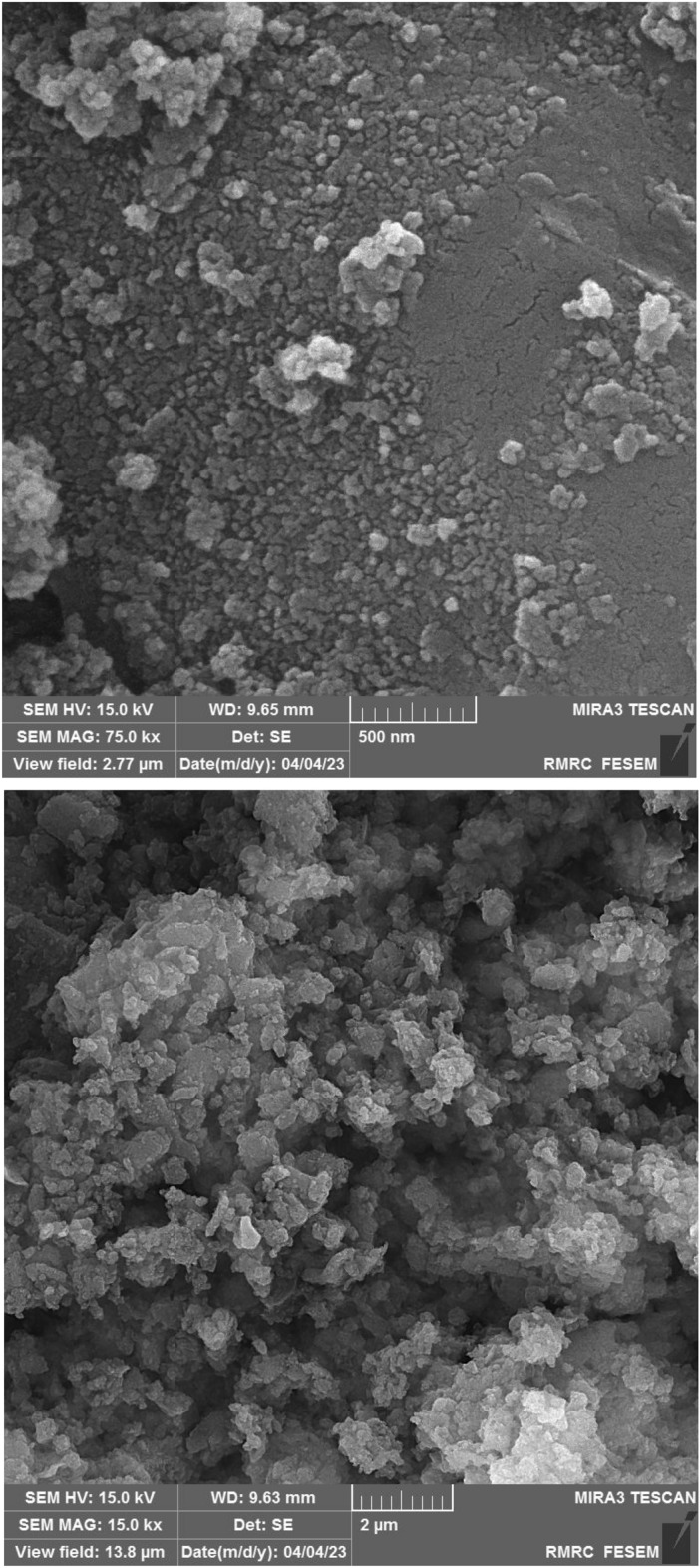
SEM image of Co(salen)@g-C_3_N_4_.

The SEM images of Co(salen)@g-C_3_N_4_ at different magnitudes were performed to support the structural and spectroscopic features, and get more information about the catalyst, and results are shown in Fig. 3. g-C_3_N_4_ was produced through direct calcination of melamine and showed a two-dimensional (2D) structure consisting of stacked thin sheets with wrinkles and irregular shapes. These sheets have noticeable micro-holes on their surfaces, adding to the material's unique characteristics. The 2D structure of g-C_3_N_4_ did not change after incorporation of Co(salen) on the surface of carbon nitride (Figure S2 in Supporting Information).

**Figure 4:**
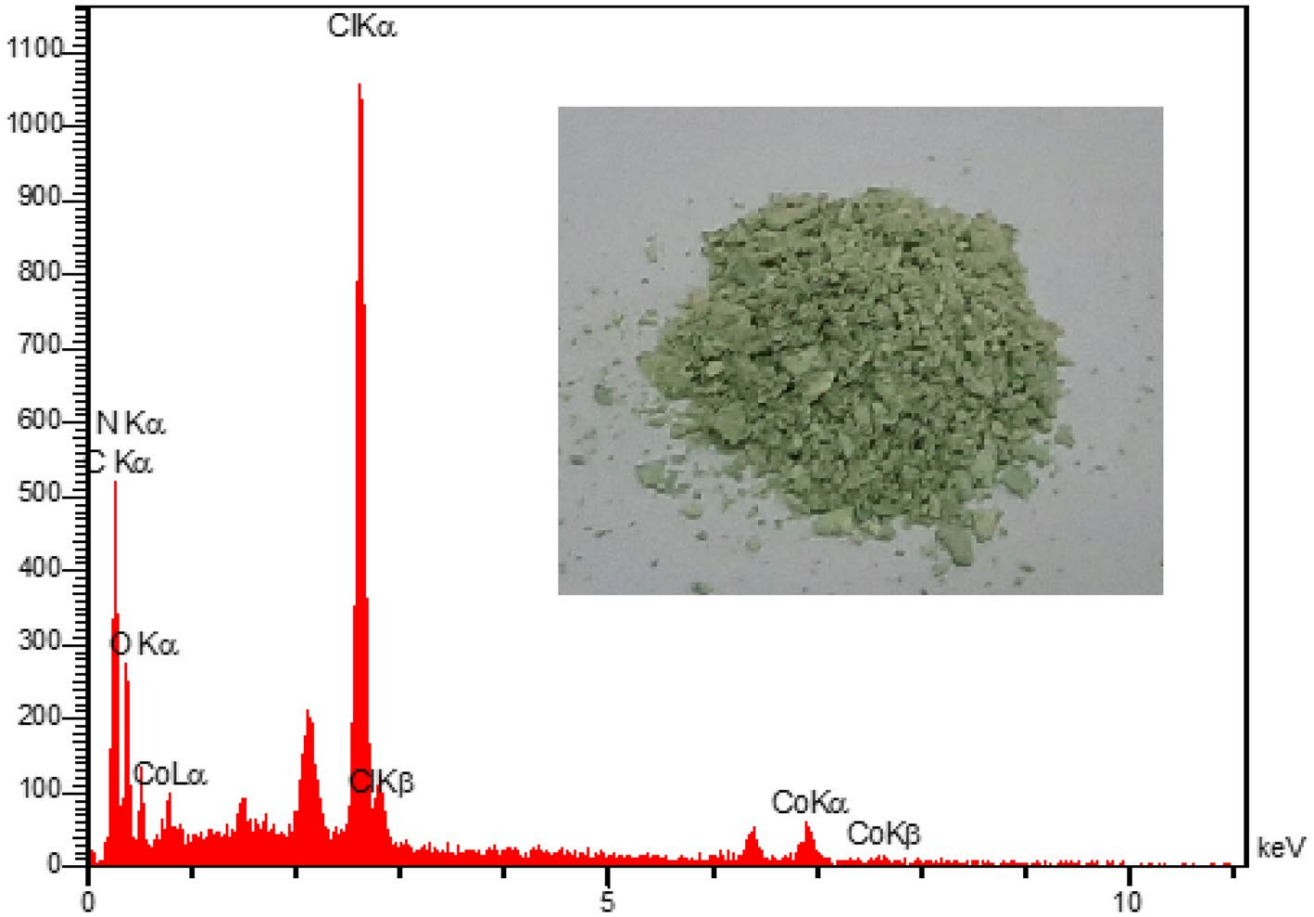
EDX pattern of Co(salen)@g-C_3_N_4_.

The EDX spectroscopy image of Co(salen)@g-C_3_N_4_ is shown in Fig. 4. The EDS spectrum provided in Fig. 4 confirms the presence of the chemical compound CoCl_2_ in the g-C_3_N_4_ catalyst. The spectrum shows the appearance of elements related to Co (cobalt), Cl (chlorine), O (oxygen), N (nitrogen), and C (carbon). This provides evidence of the presence of these elements and their compounds on the surface of Co(salen)@g-C_3_N_4_ catalyst.

**Figure 5:**
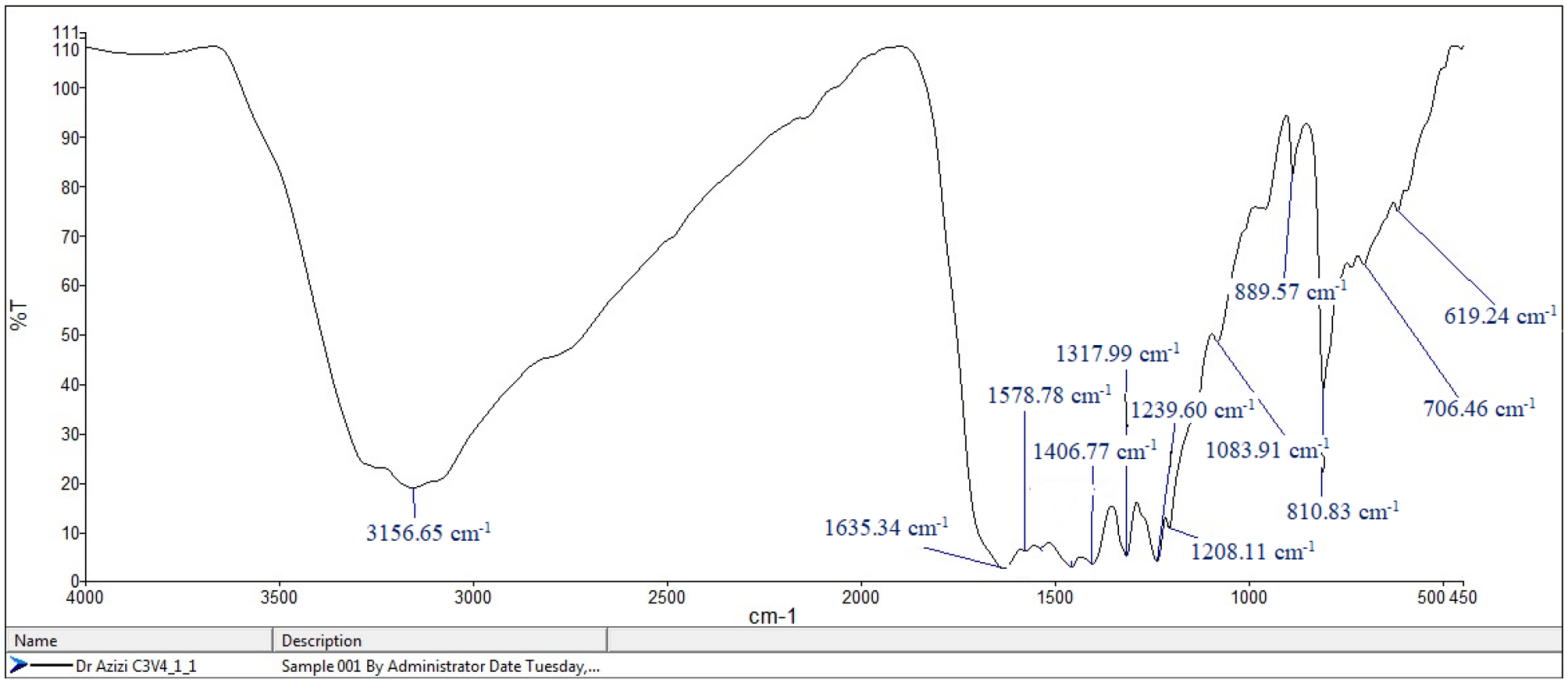
FTIR spectra of Co(salen)@g-C_3_N_4_.

The FTIR (Fig. 5) confirms the functionalization of graphitic carbon nitride surface and several characteristic absorption bands appeared in the FTIR spectrum. FTIR spectra of g-C_3_N_4_, showed typical C-N heterocycle stretches at 1251, 1325, 1450, 1578, and 1635 cm^−1^, as well as the characteristic breathing mode of triazine units at 889 cm^−1^. The broad absorption bands at 3156 cm^−1^ correspond to the stretching vibrations of hydroxyl bonds associated with absorbed water in the crystal lattice of carbon nitride. These hydroxyl bonds are physically and chemically attached to the crystalline structure of carbon nitride and manifest as a broad peak in the FTIR spectrum. Furthermore, a narrow peak at 619 cm^−1^ that is due to the intrinsic Co–O stretching vibration indicates the presence of Co in the catalyst.

**Figure 6:**
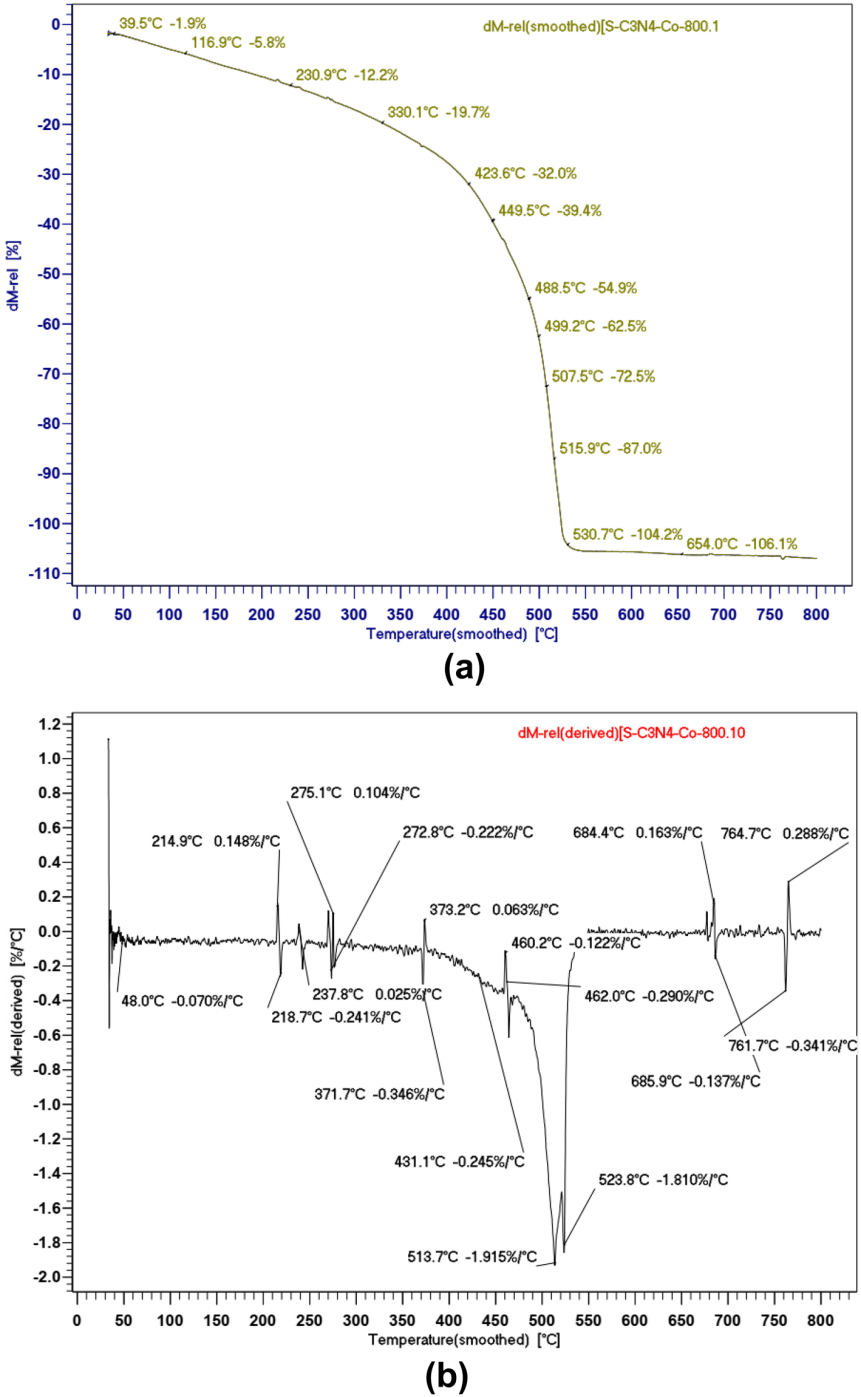
**(a)** TGA curve Co(salen)@g-C3N4. (**b)** TG curve Co(salen)@g-C_3_N_4_.

To assess the thermal stability of the catalyst, thermogravimetric analysis (TGA and TG) was conducted under an air atmosphere, ranging from 50 to 800 °C. During the TGA, the sample's weight change was monitored as it was subjected to increasing temperatures. This analysis provides valuable information about the decomposition and thermal behavior of the carbon nitride polymer, allowing for a better understanding of its stability and potential applications (Fig. 6a,b). According to the observations presented in Fig. 6a, the initial mass loss occurring below 200 °C is primarily attributed to the evaporation of adsorbed water or other volatile impurities present on the surface of the sample. As the temperature increases, the heating process leads to the decomposition of the applied salicylaldehyde and g-C_3_N_4_. This decomposition results in the chemical conversion of the salicylaldehyde into carbon-containing gases and g-C_3_N_4_ into nitrogen and carbon-containing gases. The decomposition of salicylaldehyde initiates at 200 °C and is completed by 350 °C. The main region of weight loss occurs between 350 and 550 °C, which corresponds to the decomposition of carbon nitride. At temperatures exceeding 600 °C, a residual weight of 1% is observed, which is attributed to the presence of cobalt oxide content in the catalyst.

After the preparation and characterization of the Co(salen)@g-C_3_N_4_ catalyst, its catalytic performance for the oxidation of benzaldehyde and its derivatives was investigated. The model reaction chosen for this study was the oxidation of benzaldehyde using H_2_O_2_. The goal was to optimize the yields and reaction times by varying the amount of catalyst, oxidation conditions, and temperature. The results of these optimization experiments are presented in Table 1. In the control reactions without the catalyst (Table 1, entry 1), the oxidation reaction of benzaldehyde with H_2_O_2_ did not proceed readily, resulting in low product yields. However, when metal-containing M(salen)@g-C_3_N_4_ catalysts were introduced, the oxidation reaction was promptly initiated. As shown in Table 1, the catalytic activity of the M(salen)@g-C_3_N_4_ catalysts followed the trend of Co(salen)@g-C_3_N_4_ > Mn(salen)@g-C_3_N_4_ > Cu(salen)@g-C_3_N_4_, with decreasing activity observed (Table 1, entries 2–4). The highest yield (98%) of the desired benzoic acid **2a** was achieved after 20 h of reaction at room temperature, using 3 equivalents of H_2_O_2_ under solvent-free conditions (entry 4). It should be noted that reducing the amount of H_2_O_2_ resulted in a decrease in yield (Table 1, entries 5–8). The next step involved investigating the influence of the amount of Co(salen)@g-C_3_N_4_ catalyst in the oxidation system. It was observed that increasing the amount of catalyst had a significant impact on the oxidation efficiency. As the amount of catalyst increased, the oxidation yields improved. Specifically, when the catalyst amount was 5 mg, 10 mg, 20 mg, and 40 mg, the oxidation yields were 51%, 68%, 81%, and 98%, respectively (Table 1, entries 9–12). However, the trend of oxidation yields remained stable when the amount of catalyst was further increased to 40 mg (Table 1, entry 12). The impact of reaction temperature on the rate of oxidation reactions was investigated for the model reaction in the presence of a catalyst. The experiment involved varying the temperature from room temperature to 80 °C and analyzing the resulting product yields. The reaction was carried out under optimized conditions for two hours. The results, as shown in Table 1, indicate that the yield of the product gradually increased as the reaction temperature was raised. At room temperature, the reaction rate was relatively low, suggesting that the reaction was not proceeding efficiently under these conditions. As the temperature was increased to 40 °C, the yield improved to a moderate level, indicating a faster rate of reaction. The highest yield of the product was obtained at 60 °C, where the reaction rate was optimized. This temperature provided the most favorable conditions for the oxidation reaction to occur, resulting in a high yield of the desired product. However, when the temperature was further increased to 80 °C, the yield showed a slight decrease compared to 60 °C, indicating that the reaction might have started to deviate from the optimal conditions (Table 1, entries 13–15).

**Table 1:** Optimization of reaction parameter on the model reaction.


Entry	M(salen)@g-C_3_N_4_ (mg)	Amount of H_2_O_2_ (mmol)	Time (h)	Temp (°C)	Yield (%)^a^
1	–	3	20	25	30
2	Mn(salen)@g-C_3_N_4_ (30 mg)	3	20	25	62
3	Cu(salen)@g-C_3_N_4_ (30 mg)	3	20	25	58
4	Co(salen)@g-C_3_N_4_ (30 mg)	3	20	25	98
5	Co(salen)@g-C_3_N_4_ (30 mg)	2.5	20	25	89
6	Co(salen)@g-C_3_N_4_ (30 mg)	2	20	25	78
7	Co(salen)@g-C_3_N_4_ (30 mg)	1.5	20	25	76
8	Co(salen)@g-C_3_N_4_ (30 mg)	1.0	20	25	62
9	Co(salen)@g-C_3_N_4_ (5 mg)	3	20	25	51
10	Co(salen)@g-C_3_N_4_ (10 mg)	3	20	25	68
11	Co(salen)@g-C_3_N_4_ (20 mg)	3	20	25	81
12	Co(salen)@g-C_3_N_4_ (40 mg)	3	20	25	98
13	Co(salen)@g-C_3_N_4_ (30 mg)	3	8	40	65
14^b^	Co(salen)@g-C_3_N_4_ (30 mg)	3	2	60	98 (98,98, 98)^b^
15	Co(salen)@g-C_3_N_4_ (30 mg)	5	2	80	98

^a^GC yields

^b^The reaction was repeated for three times.

The scope and generality of the oxidation reaction using aldehydes were investigated under the optimized conditions. The results are summarized in Table 2, showing the yields of benzoic acid derivatives obtained from various aldehyde derivatives. The oxidation of different aromatic and heteroaromatic aldehydes to benzoic acid derivatives was successful, with good to excellent yields. The electronic properties and steric effects of substituents on the aromatic aldehydes did not significantly influence the reaction, as the yields remained consistently high across different substituents. Aromatic aldehydes bearing both electron-withdrawing and electron-donating groups gave well to excellent yields, indicating the versatility of the reaction. Even heteroaromatic aldehydes reacted smoothly, yielding excellent results. Aldehydes containing electron-donating groups like OCH_3_ and CH_3_, provided moderate yields. The oxidation reactions were carried out at 60 °C and typically reached completion within a few hours (2–5 h). The isolated yields of the benzoic acid products were generally quantitative, indicating the efficiency of the optimized reaction conditions.

**Table 2:**
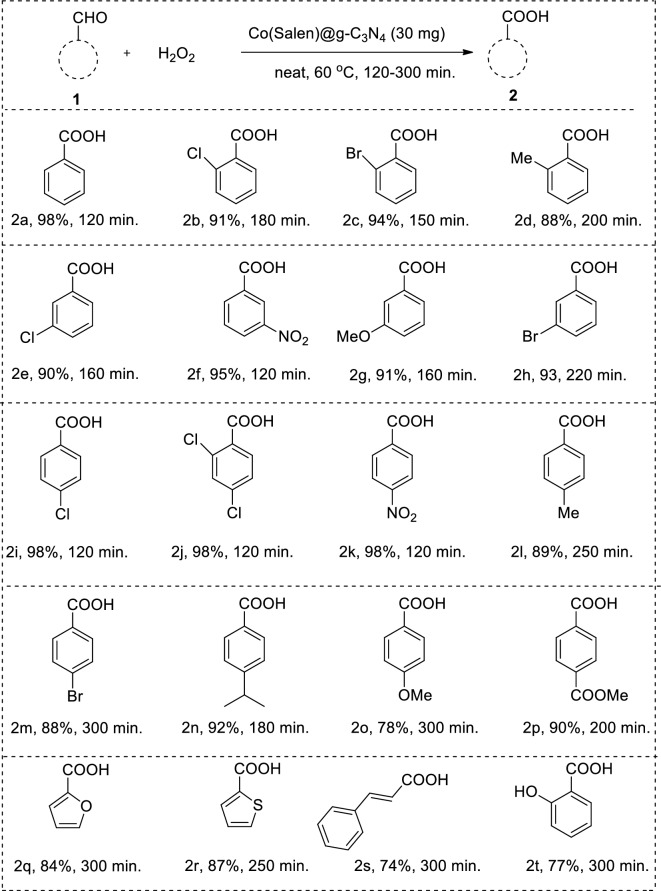
Scope and generality of the oxidation reaction using aldehydes.

The scalability of the oxidation method was evaluated by conducting a gram-scale oxidation of benzaldehyde using the optimized conditions. Remarkably, the oxidation of benzaldehyde on a 10 g scale, with a catalyst amount of only 200 mg, resulted in an impressive isolated yield of 98% for the desired benzoic acid product. This gram-scale oxidation demonstrates the practical applicability of the method and its potential for large-scale synthesis. The use of a relatively small amount of catalyst in proportion to the reaction scale further highlights the efficiency and cost-effectiveness of the process.

**Figure 7:**
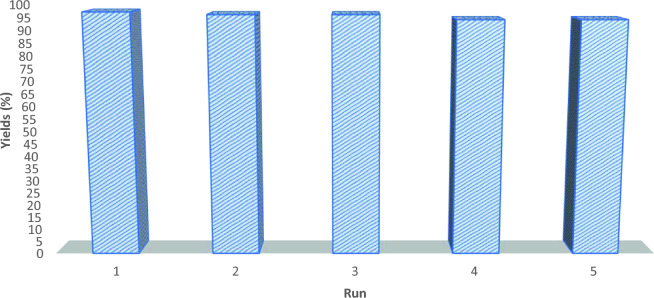
Reusability of the carbon nitride catalyst in a mole-scale reaction.

The reusability of the carbon nitride-based catalyst was investigated in a model reaction with a 5 mmol-scale reaction using 100 mg of catalyst. The results are summarized in Fig. 7, showing the performance of the recovered catalyst in successive runs. In the first and second runs (Fig. 7, entries 1 and 2), the recovered catalyst exhibited excellent reusability, maintaining its efficiency with high product yields. This indicates the stability and robustness of the catalyst, allowing for multiple reaction cycles without significant loss in performance. However, in the third and fourth runs (Fig. 7, entries 3 and 4), a slight decrease in product yield was observed. Overall, the carbon nitride catalyst showed good reusability up to the five-run, with high product yields.

A comparison was made between the prepared Co(salen)@g-C_3_N_4_ catalysts for the oxidation of benzaldehyde to benzoic acid with other reported procedures. The comparison highlighted the advantages of the proposed methodology in terms of reaction times, yields, and the environmentally friendly nature of the process. The results in Table 3 demonstrate that the Co(salen)@g-C_3_N_4_ catalysts exhibit shorter reaction times, excellent yields, and importantly, offer a greener alternative compared to other methodologies^58–64^.

**Table 3:** Comparison of literature for oxidation of benzaldehyde.

Entry	Reaction condition	Solvent	Temp (°C)	Time (h)	Yield (%)	References
a	H_3_PW_12_O_40_, Al-MCM-48, H2O2	Neat	80	6	81	^58^
b	[MoO_3_(trz)0.5], H_2_O_2_	Water	70	24	80	^59^
c	MOF-Zn-NHC, Et_3_N, O_2_	Water	100	3.5	80	^60^
d	AuNPs/BPy-PMO, NaHCO_3_, O_2_	Water	30	2	87	^61^
e	KBrO_3_/KBr, HCl	Water	100	3	50	^62^
f	Oxone	DMF	rt	3	97	^38^
g	Ni(OAc)_2_, O_2_, 6205 Torr	Ethanol/Water	20	0.5	80	^53^
h	Whole-cell biocatalyst	KPi	35	12	99	^40^
i	Geopolymer supported CuO	Neat	80	10	67	^63^
j	hydroxycyclohexylphenylketon	NaOH/DME	80	3	99	^42^
k	Co(salen)@g-C_3_N_4_, H_2_O_2_	Neat	60	2	98	This work

**Figure 8:**
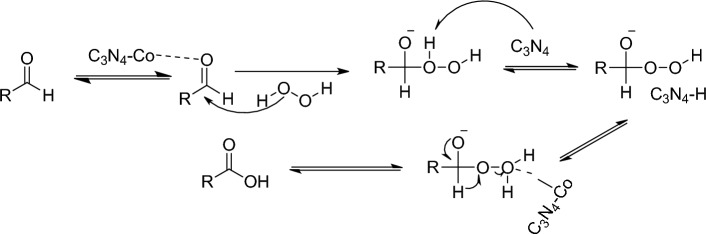
Proposed mechanism.

Based on the available data and previous reports^64^, a mechanistic proposal for the oxidation of aldehyde to acid catalyzed by cobalt can be outlined (Fig. 8). In this proposed mechanism, cobalt acts as a catalyst to facilitate the reaction. Initially, the cobalt catalyst undergoes coordination and activation of aldehyde and H_2_O_2_. H_2_O_2_ acting as a nucleophile, adds to the activated aldehyde with the assistance of the cobalt catalyst, and carbon nitride facelifted the abstraction of hydrogen from H_2_O_2_ and the formation of water. It is worth noting that a radical mechanism may also be involved in cobalt-catalyzed reactions, although further investigation is required to elucidate the precise details^64^.

## Conclusion

In this study, a novel series of reusable M(salen)@g-C_3_N_4_ catalysts (M=Co, Cu, Mn) was synthesized by incorporating metal complexes (salen) onto the g-C_3_N_4_ host. These catalysts exhibited exceptional performance for the oxidation of aldehyde derivatives in the presence of H_2_O_2_ at mild reaction conditions with short reaction times. Among them, the Co(salen)@g-C_3_N_4_ catalyst demonstrated the best catalytic activity and was further optimized for oxidation conditions. The catalysts displayed high efficiency, durability, and recyclability, making them suitable for long-term operations. The method also proved to be robust and reliable, providing a valuable approach for synthesizing benzoic acid derivatives on a larger scale. Moreover, the scalability of the method was demonstrated by achieving high isolated yields on a gram scale, indicating its robustness and reliability for synthesizing larger quantities of benzoic acid derivatives.

### Supplementary Information

Supplementary Information.

## Data Availability

The data that support the findings of this study are available on request from the corresponding author.
