# Supplementary material for: Metallosalen modified carbon nitride a versatile and reusable catalyst for environmentally friendly aldehyde oxidation
Source: Sci Rep. 2024 Apr 11;14:8498. doi: 10.1038/s41598-024-58946-3 (PMC11009278; doi:10.1038/s41598-024-58946-3)
Supplement: Supplementary file 1 — Supplementary Information. [file 41598_2024_58946_MOESM1_ESM.pdf]

Supporting information

Metallosalen Modified Carbon Nitride a Versatile and Reusable Catalyst for Environmentally Friendly Aldehyde Oxidation

Reza Eskandari Sedighi[a], Mahdi Behzad[a*], Najmedin Azizi[b*]

[a] Faculty of Chemistry, Semnan University, Semnan, Iran

[b] Chemistry and Chemical Engineering Research Center of Iran, P.O. Box 14335-186, Tehran, Iran

Email: mbehzad@semnan.ac.ir, azizi@ccerci.ac.ir

Key Findings:

- The synthesized M(salen)@g-C3N4 catalysts (M = Co, Cu, Mn) demonstrated exceptional performance in the oxidation of aldehyde derivatives in the presence of H2O2 under mild reaction conditions and with short reaction times.
- The Co(salen)@g-C3N4 catalyst exhibited the highest catalytic activity among the studied catalysts and was further optimized for oxidation conditions.
- The catalysts showed high efficiency, durability, and recyclability, making them suitable for long-term operations.
- The method proved to be robust and reliable for synthesizing benzoic acid derivatives on a larger scale, as demonstrated by high isolated yields on a gram-scale.

Hypothesis, New Concepts, and Innovations:

- The innovation lies in the synthesis of reusable M(salen)@g-C3N4 catalysts, where metal complexes (salen) are incorporated onto the g-C3N4 host.
- The incorporation of metal complexes onto the g-C3N4 host enhances the catalytic activity and stability of the catalysts, leading to exceptional performance in the oxidation of aldehyde derivatives.
- The use of H2O2 as an oxidant under mild reaction conditions and short reaction times provides a more environmentally friendly and efficient approach for the oxidation reactions.
- The study introduces a valuable approach for synthesizing benzoic acid derivatives on a larger scale, highlighting the scalability of the method.

Key Improvements:

- Compared to previous studies, the M(salen)@g-C3N4 catalysts showed superior catalytic activity and recyclability. This is an improvement over traditional catalysts used for oxidation reactions.
- The method presented in the study offers a more efficient and environmentally friendly approach by utilizing H2O2 as an oxidant, avoiding the need for toxic or hazardous reagents typically employed in oxidation reactions.
- The scalability of the method was demonstrated by achieving high isolated yields on a gram-scale, indicating its robustness and reliability for synthesizing larger quantities of benzoic acid derivatives.

Vision for Future Work:

- Further optimization of the Co(salen)@g-C3N4 catalyst and exploration of its application in other oxidation reactions to expand its scope and potential industrial applications.

- Investigation of the reaction mechanism and understanding the role of the M(salen)@g-C3N4 catalysts in the oxidation process to provide insights for further catalyst design and improvement.
- Exploration of the potential of the M(salen)@g-C3N4 catalysts in other catalytic reactions beyond oxidation, such as hydrogenation or carbon-carbon bond formation.
- Investigation of the stability and performance of the catalysts under various reaction conditions, including different substrates and reaction parameters, to establish their versatility and potential limitations.
- Exploration of other metal complexes and hosts for catalyst design to expand the range of reusable catalysts with enhanced catalytic activity and stability.

Experimental

2.1. Materials and Equipment

2.1.1. Chemicals

All chemicals and reagents were analytically pure, and they were purchased from Merck company. The required chemicals for the synthesis of the three catalysts including cobalt chloride ($CoCl_2$), copper chloride ($CuCl_2$), manganese chloride ($MnCl_2$), salicylaldehyde, methanol, and ethanol as well as hydrogen peroxide (H_2O_2) and aldehyde derivatives were commercially available. Carbon nitride was prepared according to a reported procedure in the literature.

2.1.2. Instruments and Equipment

Various instruments were employed for characterization and analysis of catalyst. The melting points of compounds were measured using the Buchi 535 melting-point apparatus. The progress of the reactions was monitored through thin-layer chromatography (TLC) using TLC plates. UV light was employed as the detecting agent, and the separation and movement of compounds were visualized under UV light. SEM and EDS spectra were obtained using a TESCAN Vega3 Model scanning electron microscope (SEM). Powder X-ray diffraction (XRD) analyses were performed using a Bruker AXS-D8 Advance diffractometer. FT-IR spectroscopy was conducted using a Shimadzu IR-460 instrument.

2.2. Preparation of g-C_3N_4

The synthesis of bulk g-C_3N_4 involved a direct heating method using melamine in air, following the procedure outlined in our previous paper [23]. 20 g of melamine powder was placed into a covered 50 mL alumina crucible and was subjected to gradual heating in a muffle furnace. The temperature was increased at a rate of 5°C per minute until reaching a final temperature of 550°C and maintained at 550°C for a duration of 4 hours. After the reaction time, the crucible was allowed to cool down naturally to room temperature. A light-yellow powder, which corresponds to the synthesized bulk g-C_3N_4, was obtained and collected from the crucible. g-C_3N_4 nanosheets was prepared through thermal exfoliation. The 5 g of bulk g-C_3N_4 was placed into an uncovered crucible. The crucible with the bulk g-C_3N_4 was subjected to heat treatment in a furnace at a temperature of 550°C for a duration of 3 hours. After the heat treatment, a white powder, consisting of g-C_3N_4 nanosheets, was obtained.

2.3. Preparation of Co(salen)@g-C_3N_4

1 g of g-C_3N_4 was added to a dry ethanol solution (50 mL) and the mixture was ultrasound with ultrasonic probe in 10 min to ensure proper dispersion of g-C_3N_4 in ethanol. To the g-C_3N_4 suspension, 0.5 g of salicylaldehyde was added. The resulting suspension was refluxed for 24 hours with constant stirring. The solution was allowed to cool to room temperature. It was then washed twice with 15 mL portions of ethanol. 1.0 g of salen@g-C_3N_4 was dispersed in 50 mL of dry ethanol and 0.5 g of cobalt(II) chloride was added. The resulting solution was stirred at reflux for 8 hours. After the refluxing period, the solid product was washed twice with 15 mL portions of ethanol and dried (Figure 1).

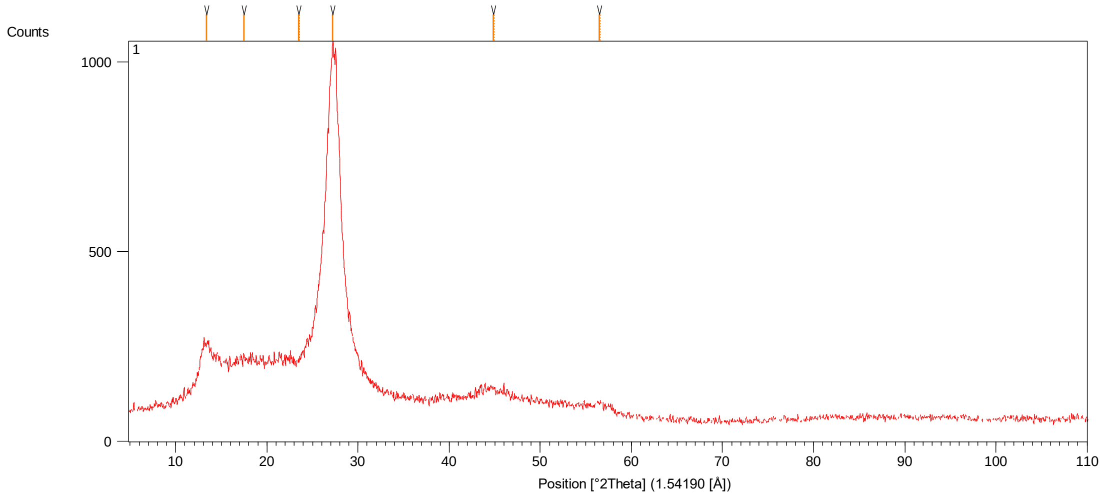

Figure S1. XRD pattern of g-C_3N_4

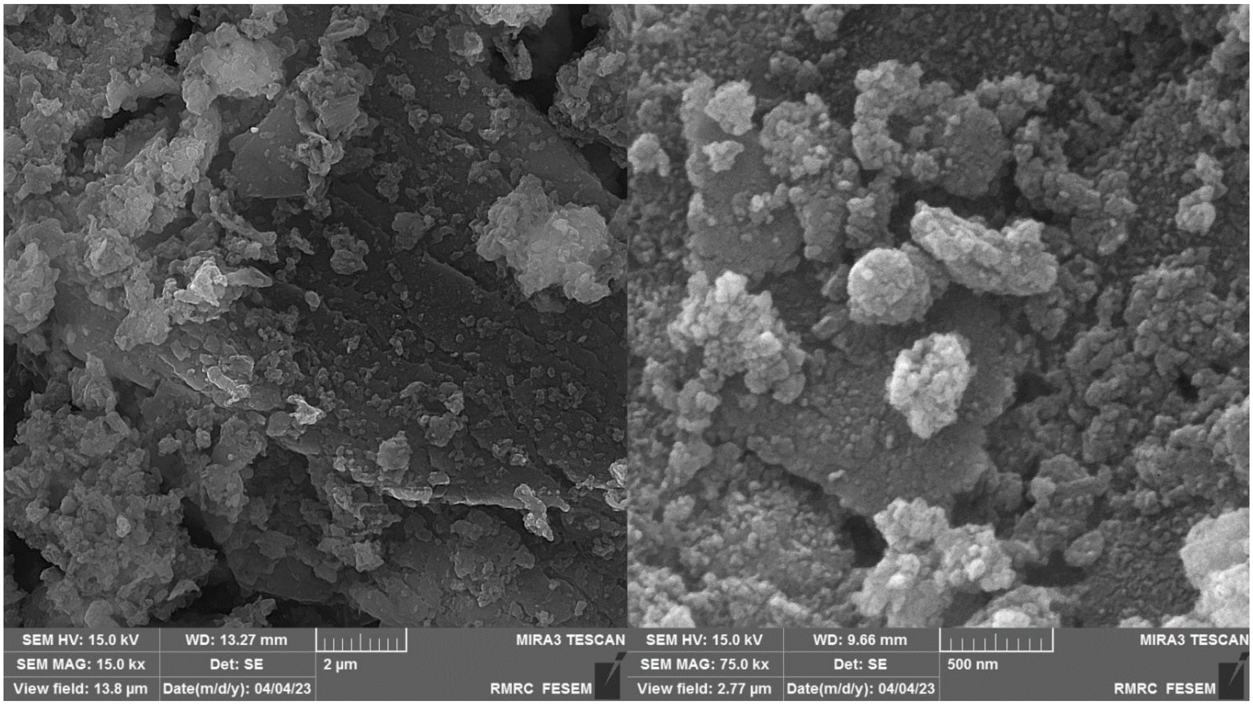

Figure S2. SEM image of g-C_3N_4

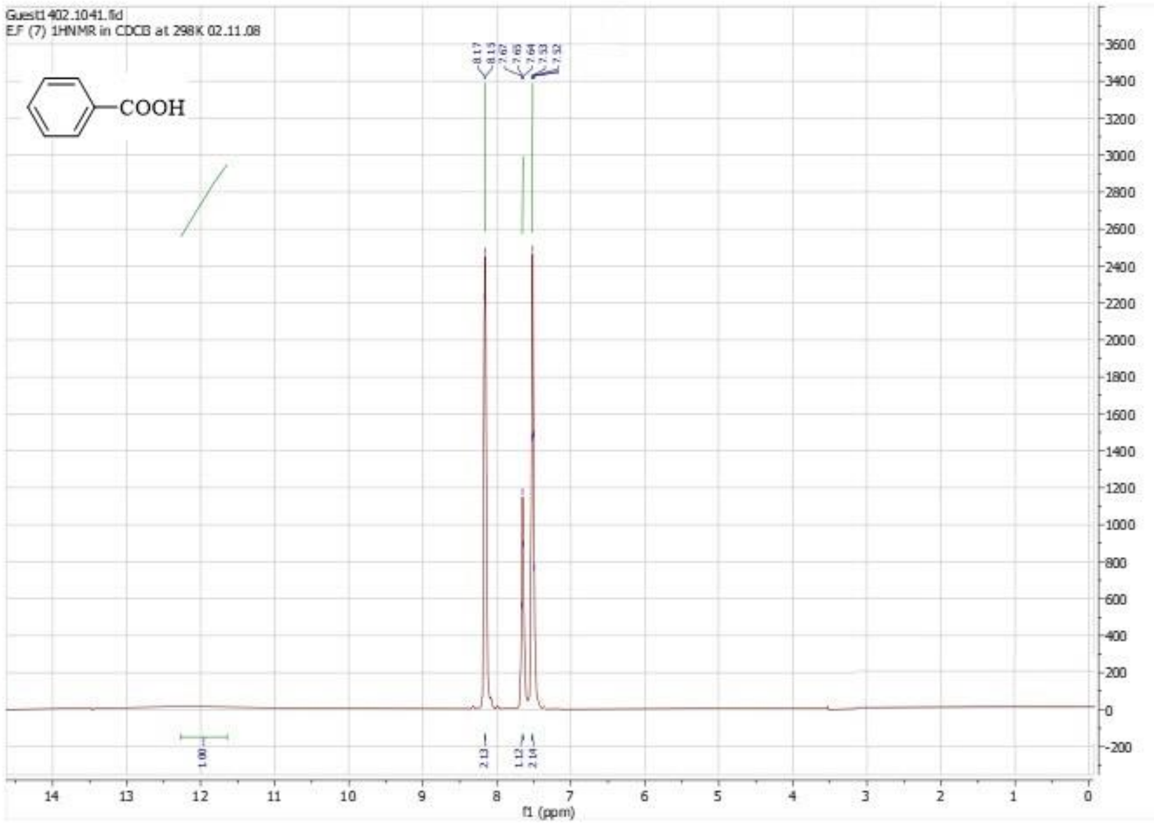

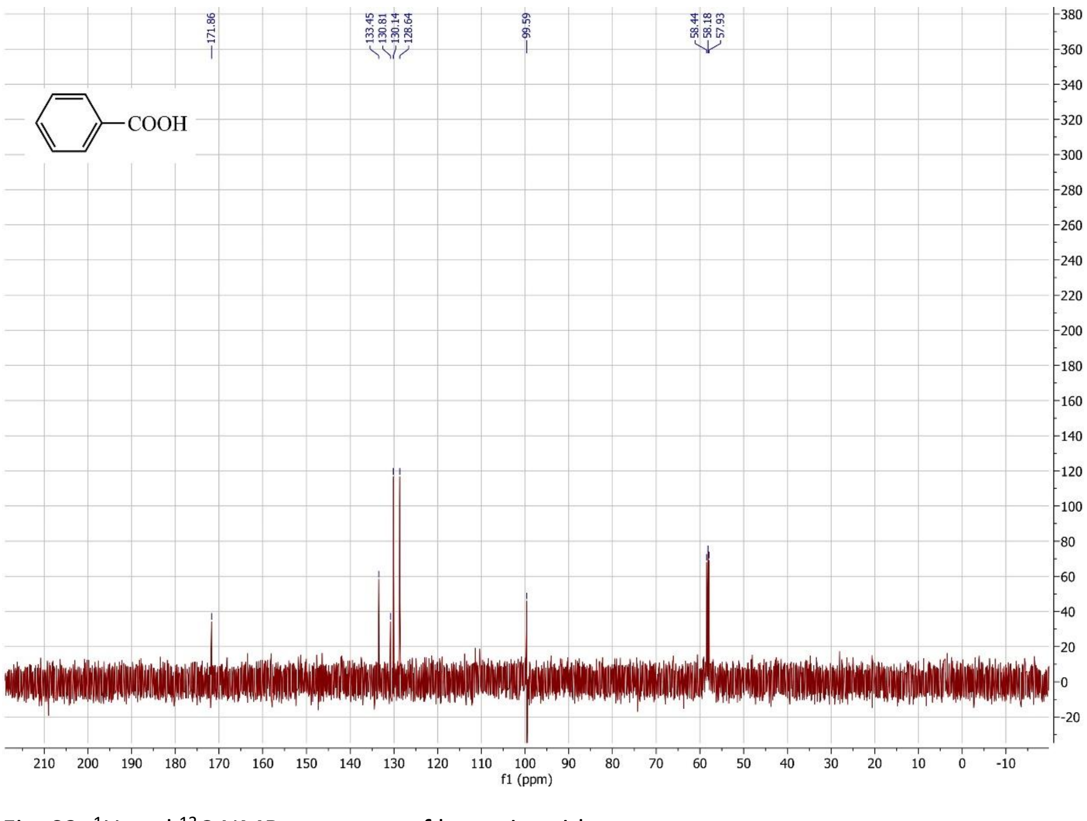

Fig. S3: ^{1}H and ^{13}C NMR spectrum of benzoic acid

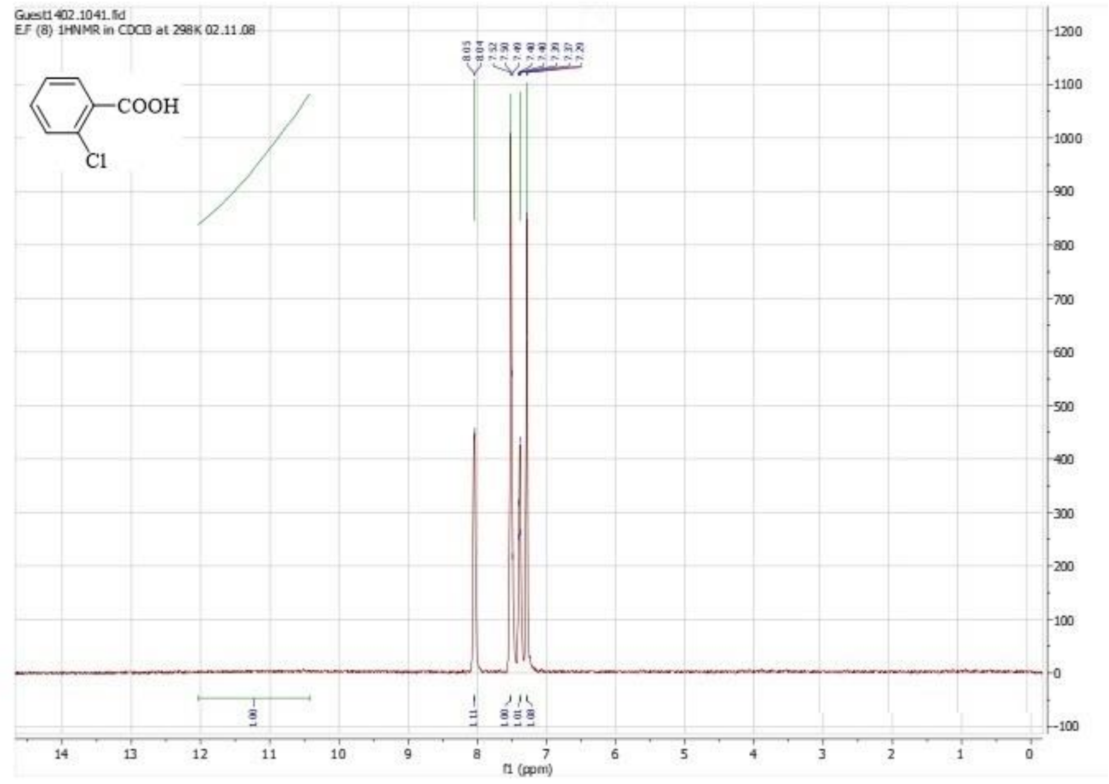

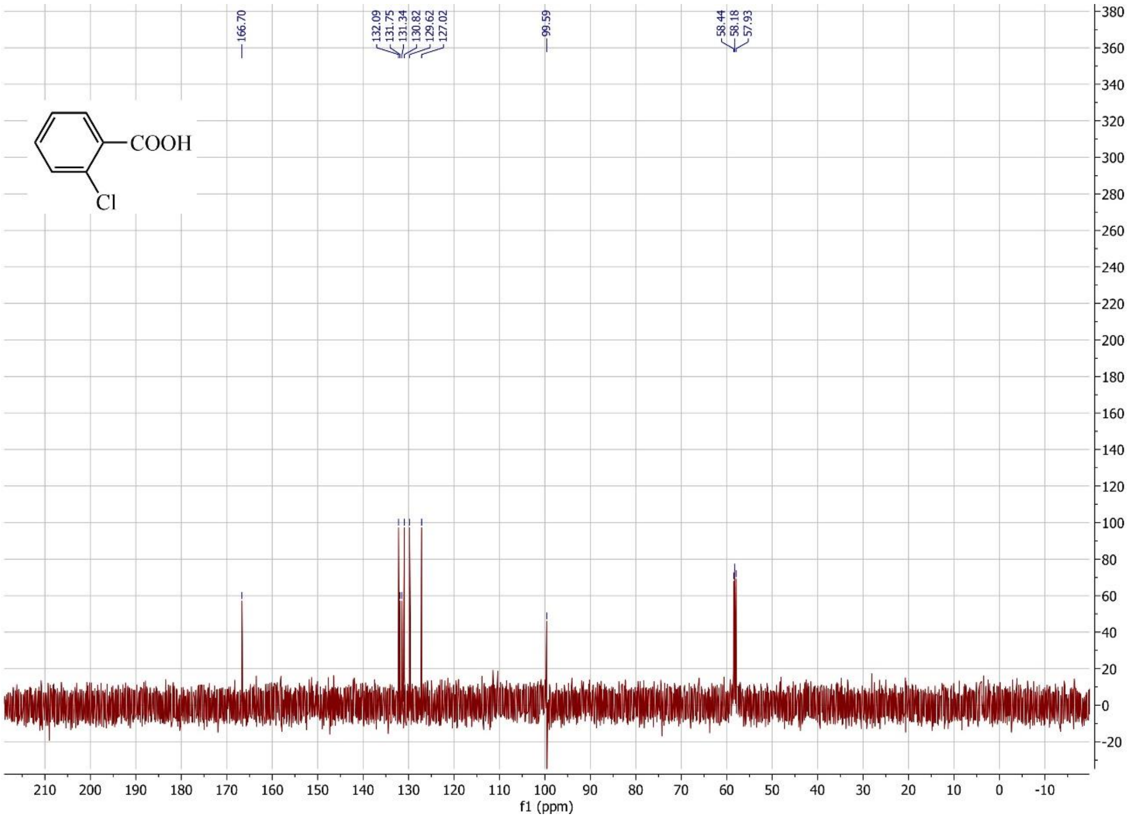

Fig. S4: ^{1}H and ^{13}C NMR spectrum of 2-Chlorobenzoic acid

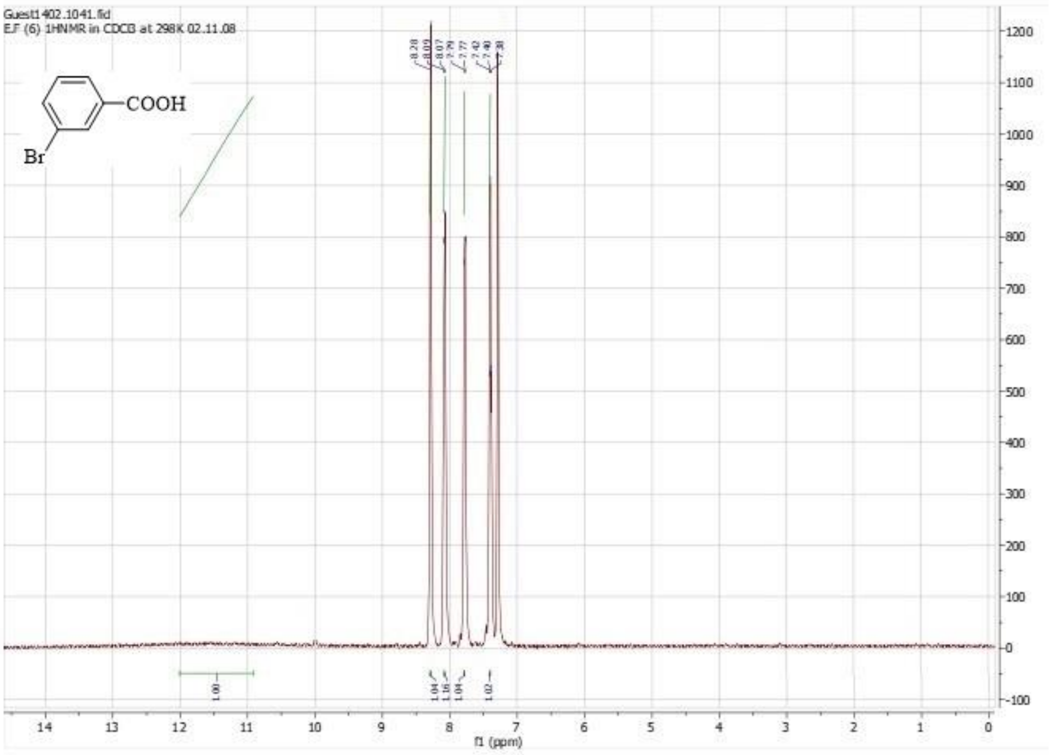

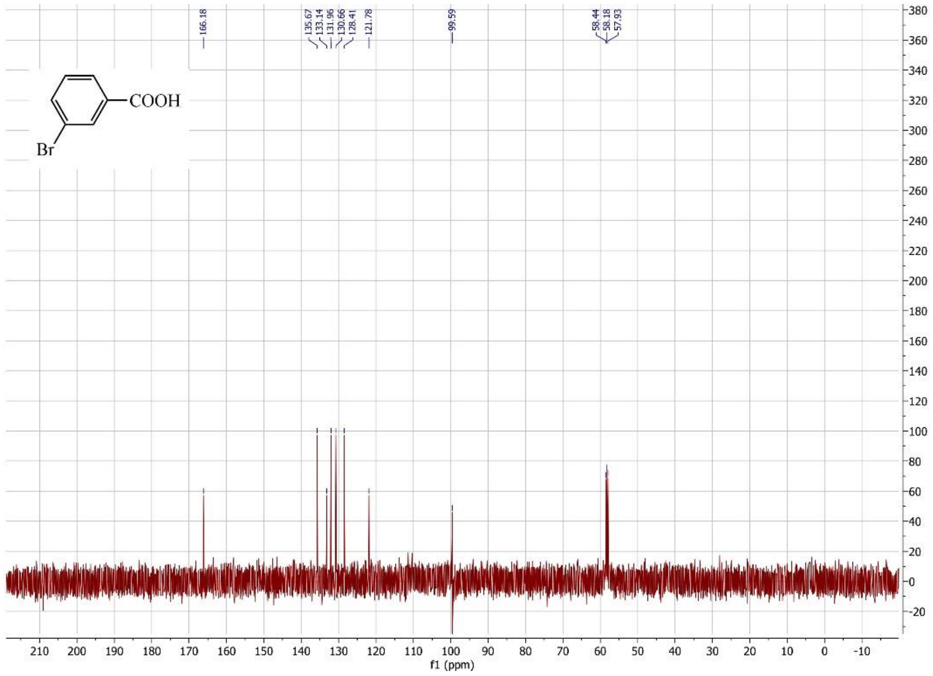

Fig. S5: ^{1}H and ^{13}C NMR spectrum of 3-bromobenzoic acid

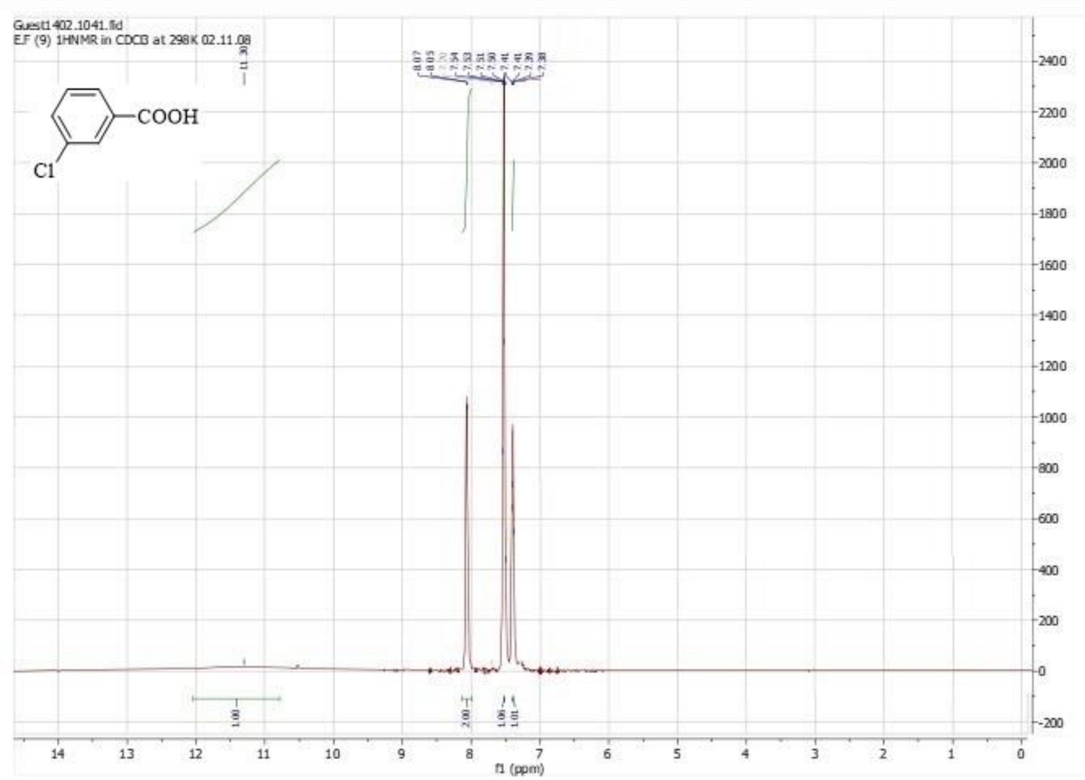

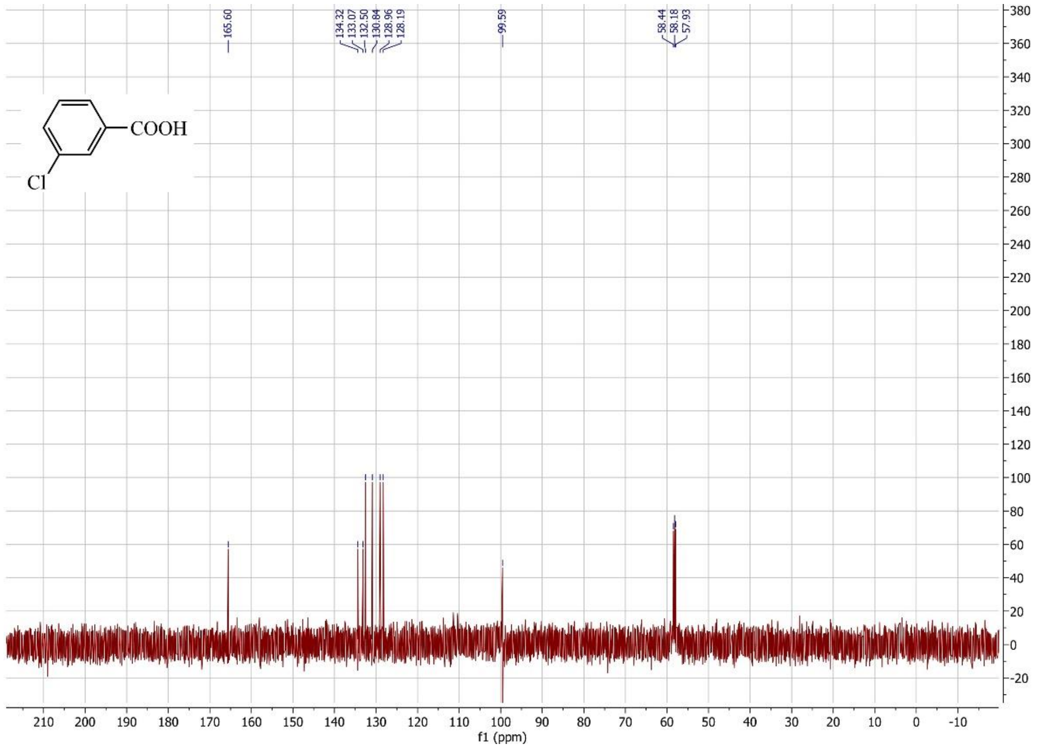

Fig. S6: ^{1}H and ^{13}C NMR spectrum of 3-Chlorobenzoic acid

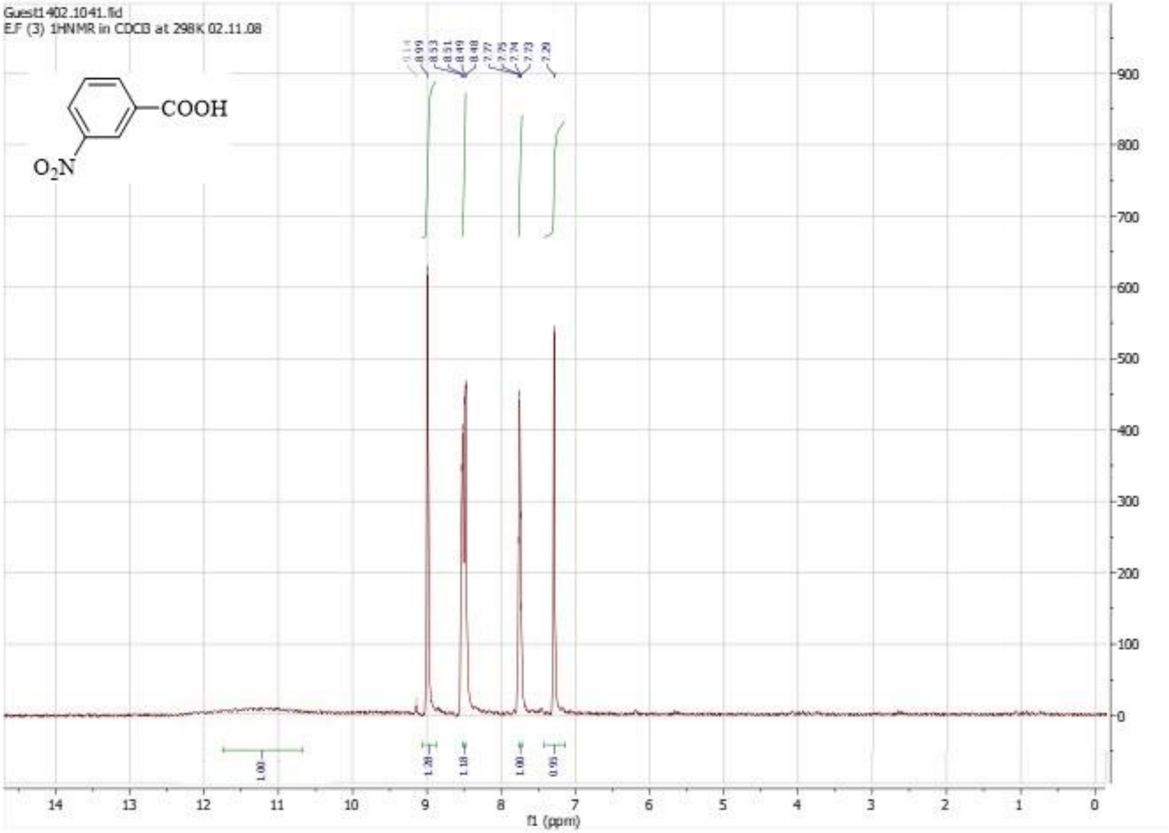

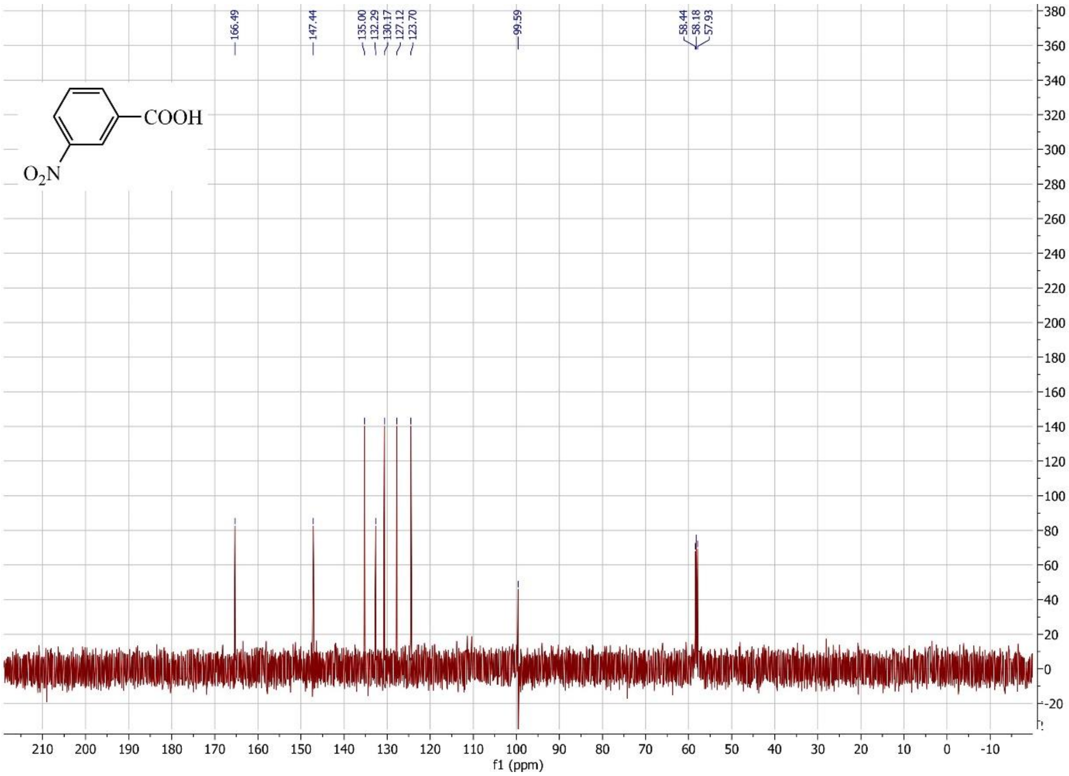

Fig. S7: ^{1}H and ^{13}C NMR spectrum of 3-Nitrobenzoic acid

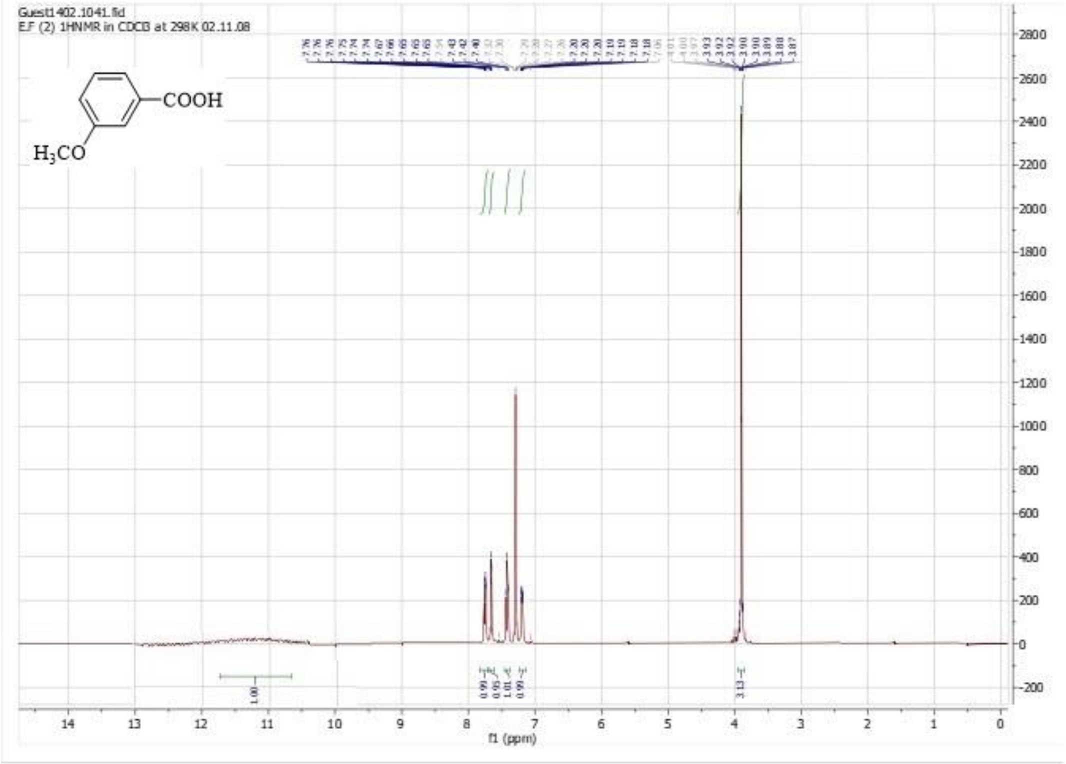

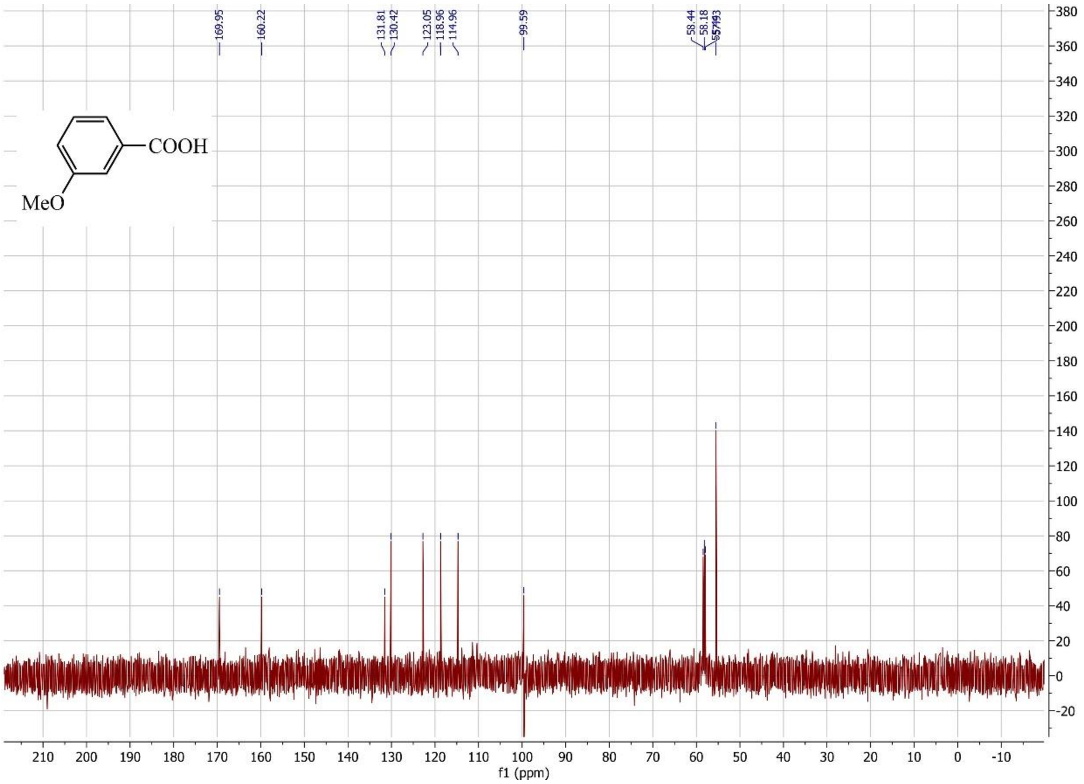

Fig. S8: ^{1}H and ^{13}C NMR spectrum of 3-Methoxybenzoic acid

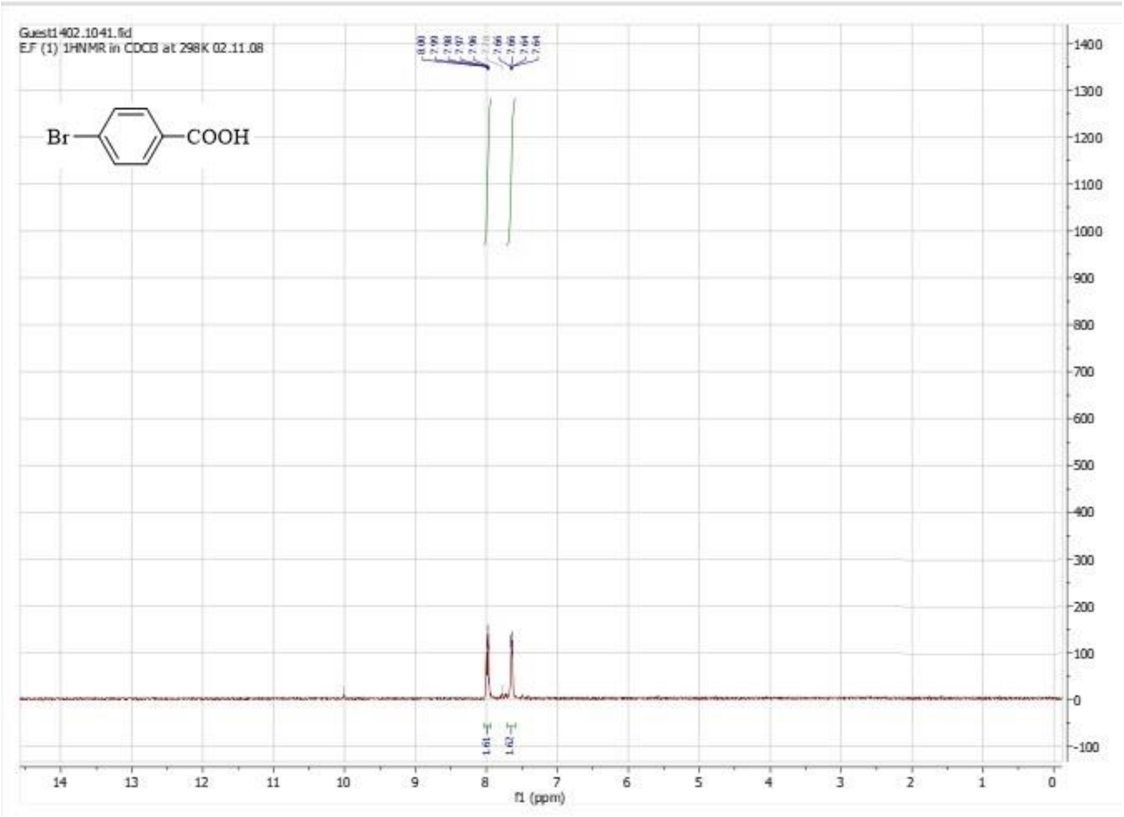

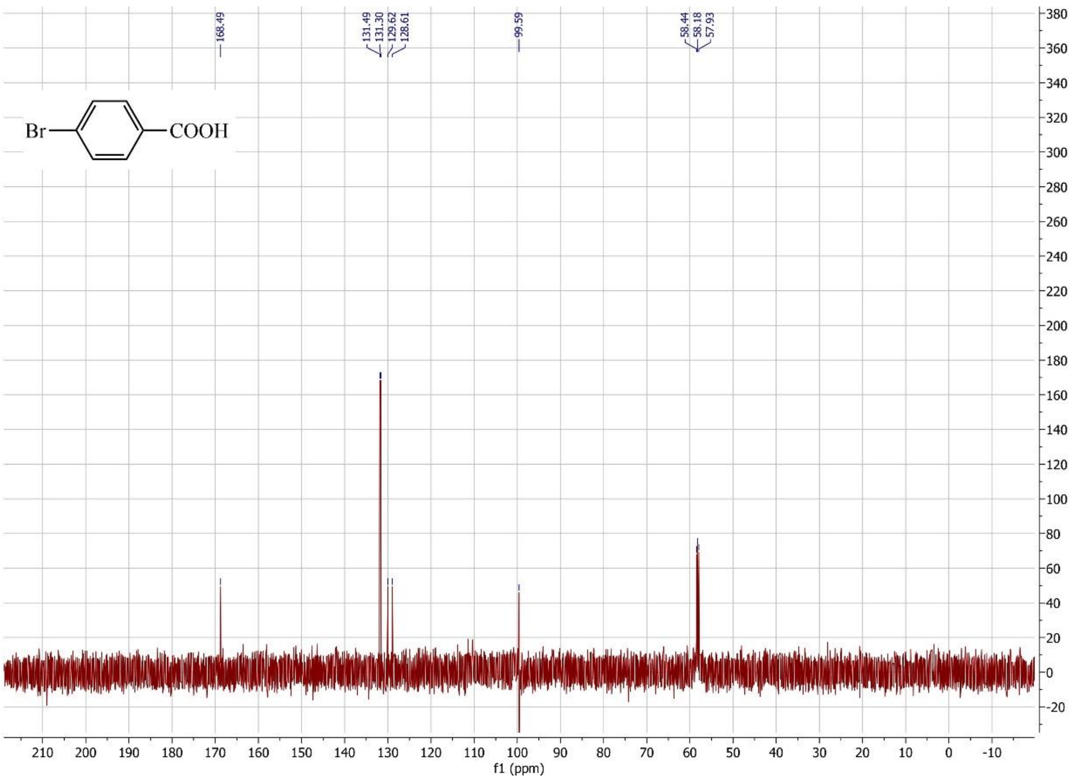

Fig. S9: ^{1}H and ^{13}C NMR spectrum of 4-bromobenzoic acid

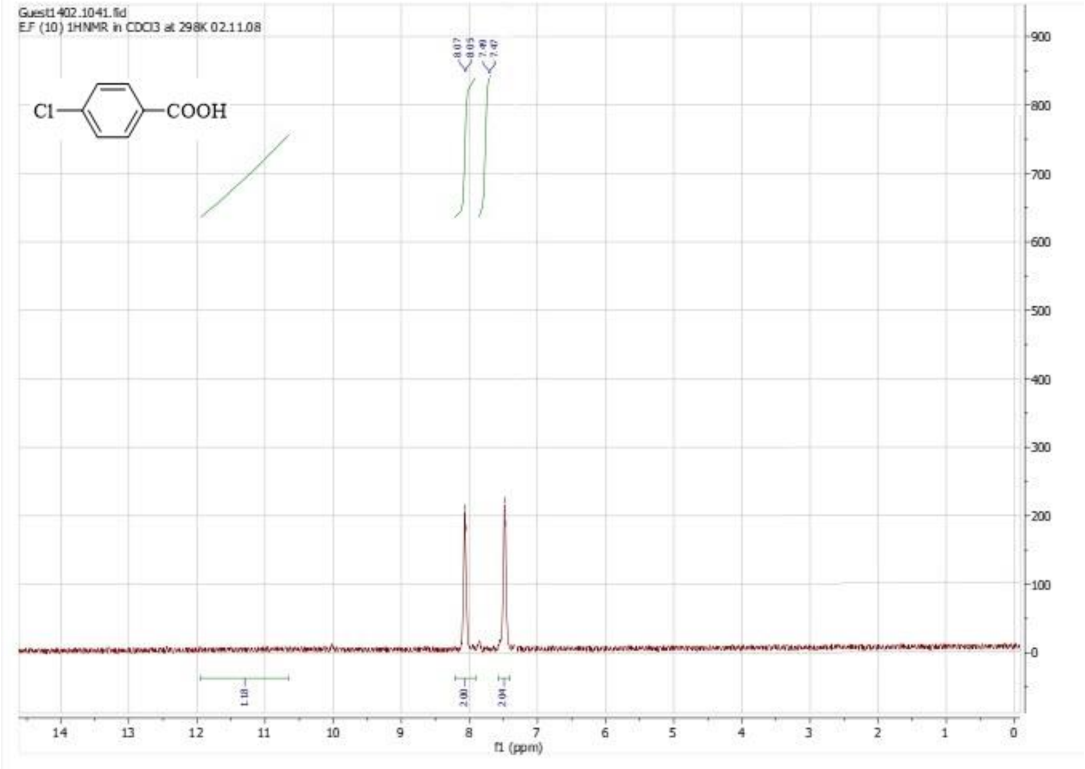

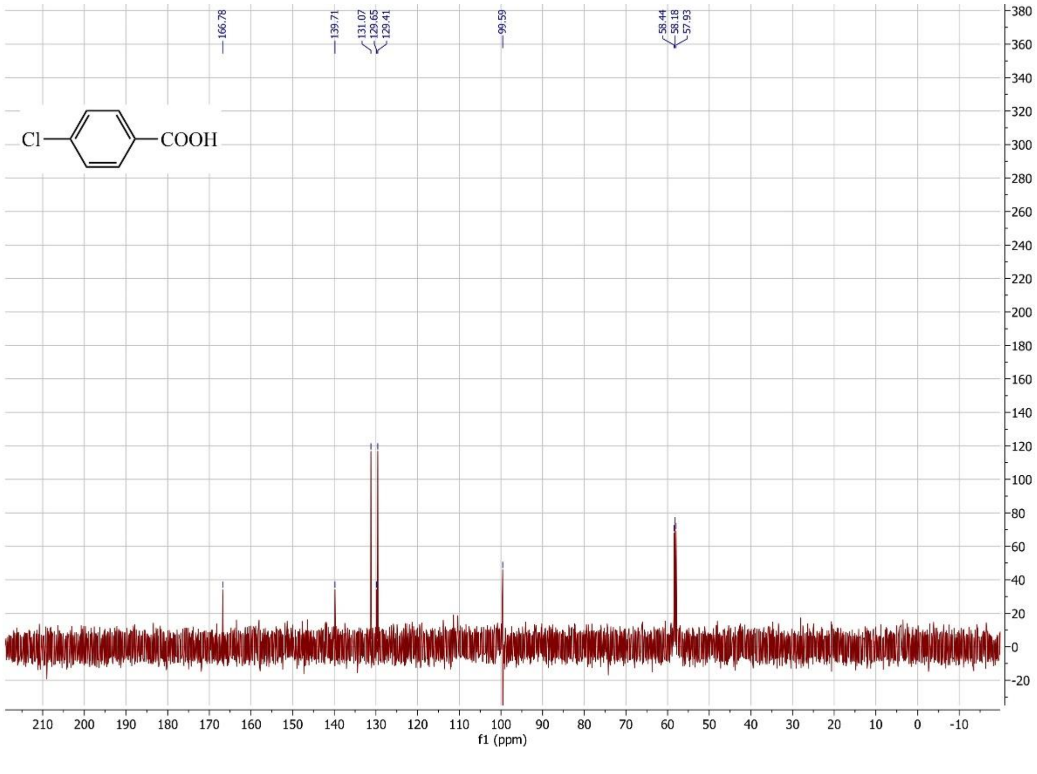

Fig. S10: ^{1}H and ^{13}C NMR spectrum of 4-Chlorobenzoic acid

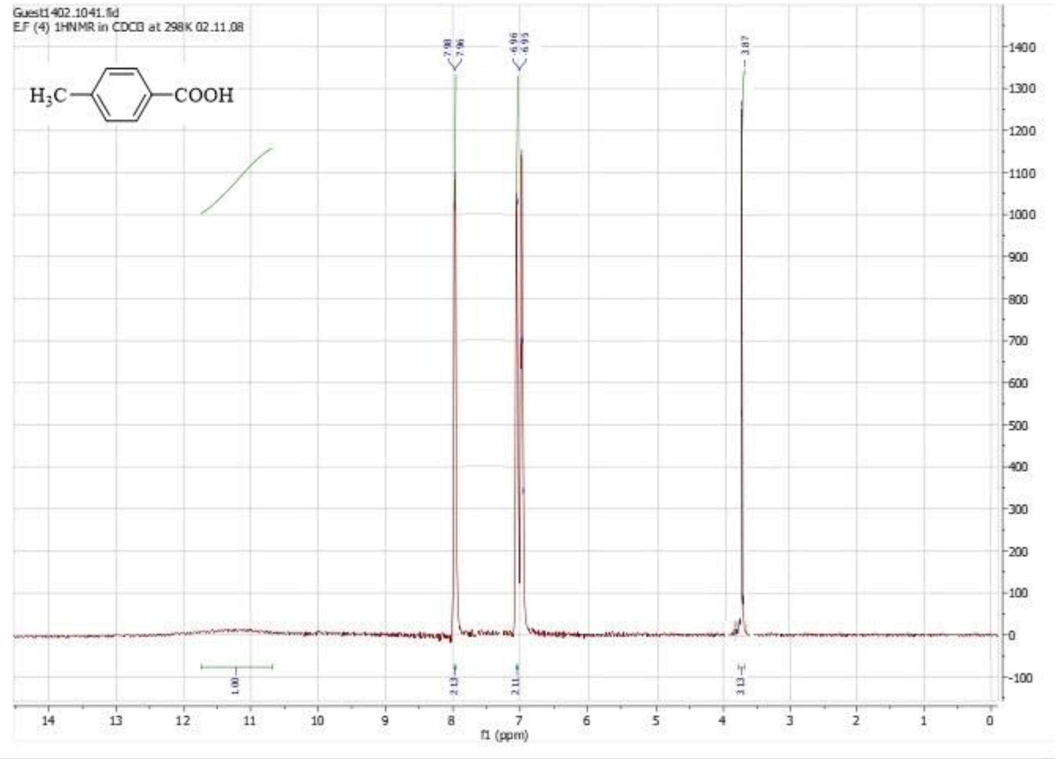

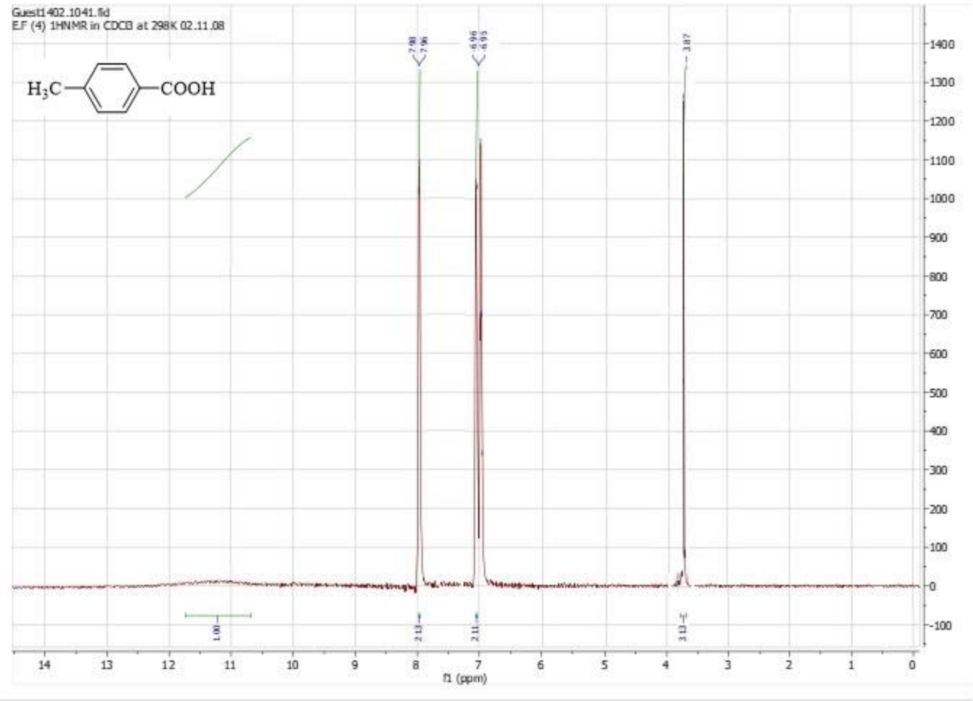

Fig. S11: 1H and 13C NMR spectrum of 4-Methylbenzoic acid

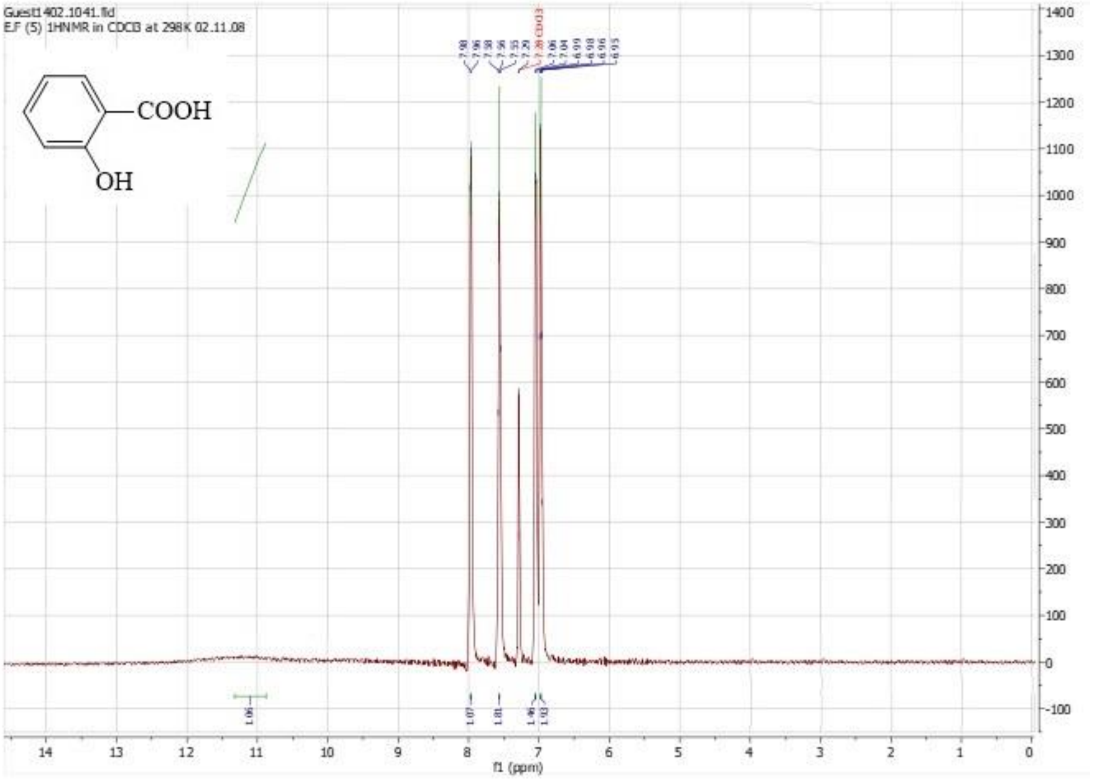

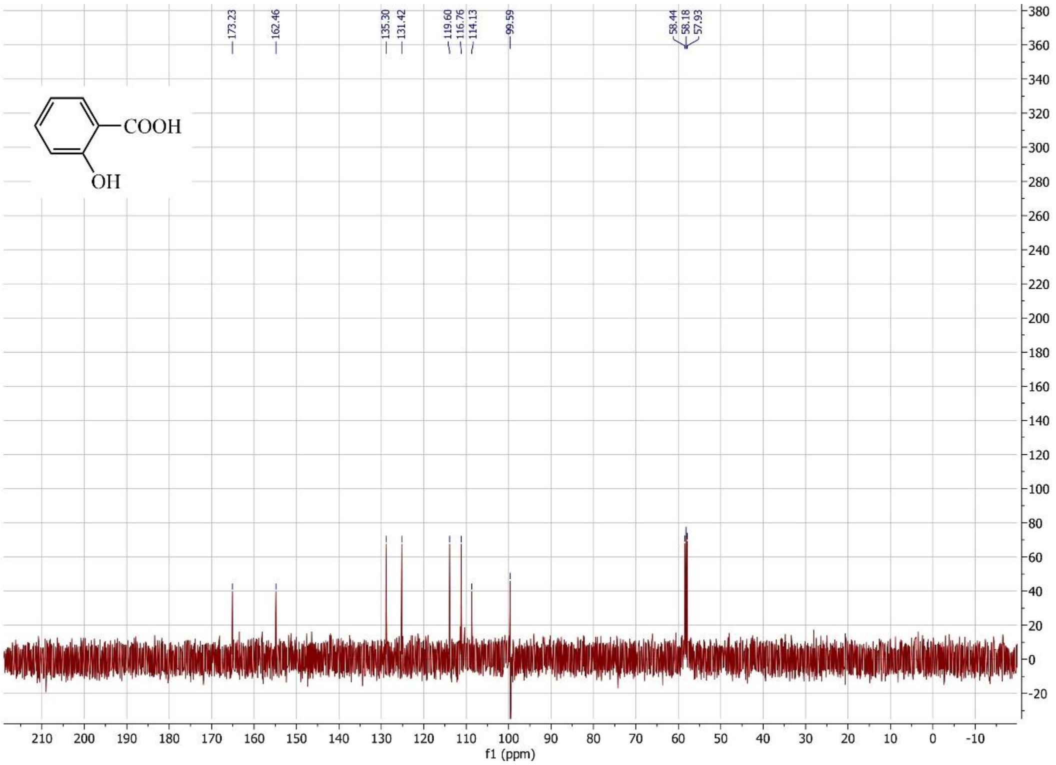

Fig. S12: ^{1}H and ^{13}C NMR spectrum of Salicylic acid

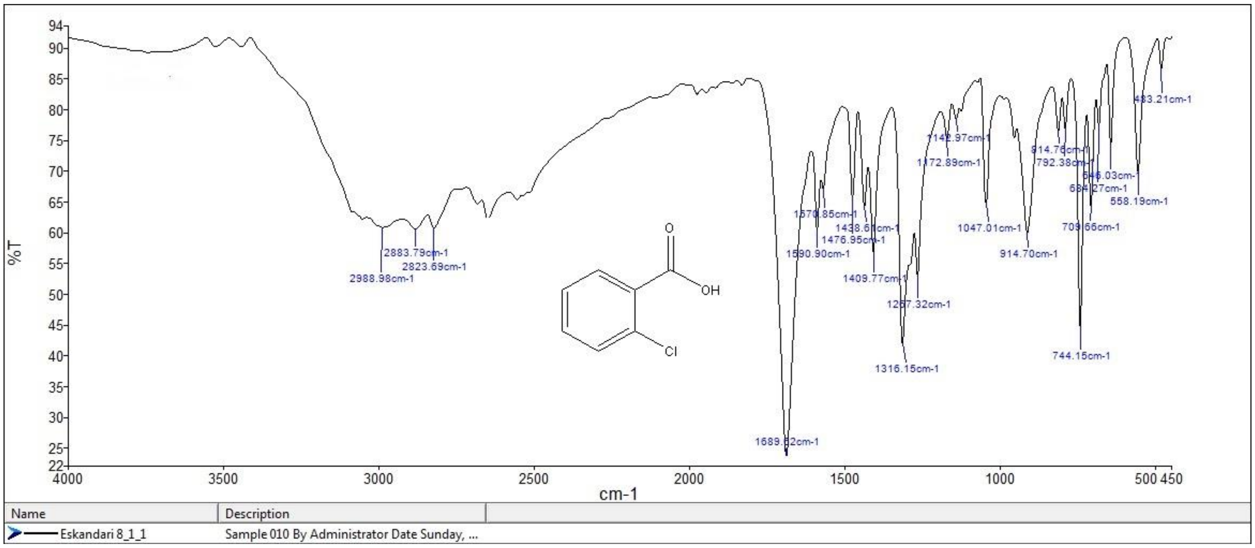

Fig. S13: FTIR spectrum of 2-Chlorobenzoic acid

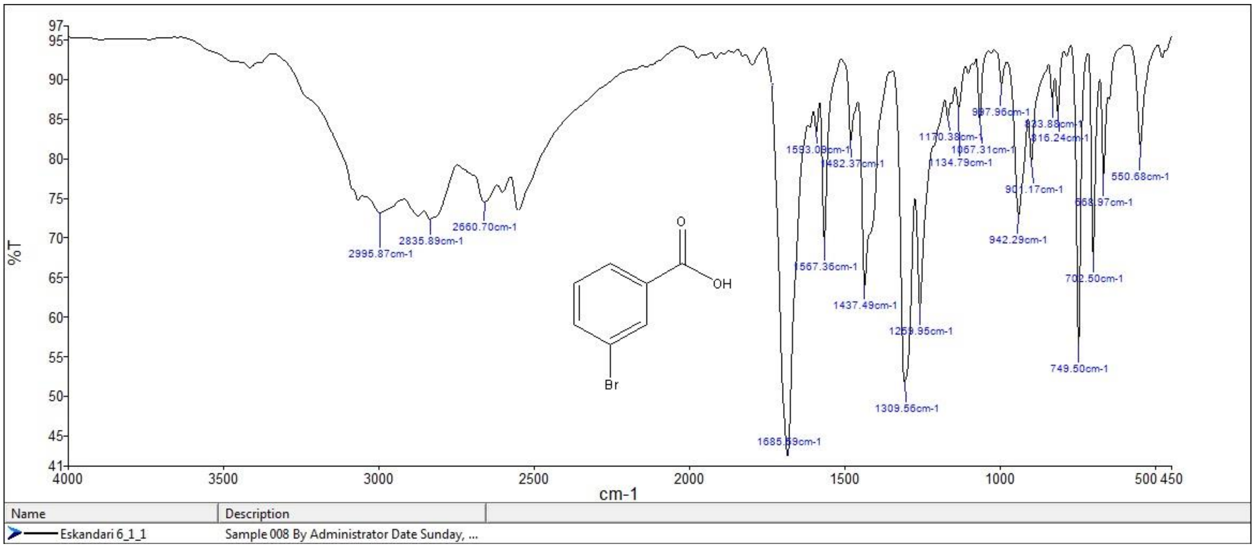

Fig. S14: FTIR spectrum of 3-Bromobenzoic acid

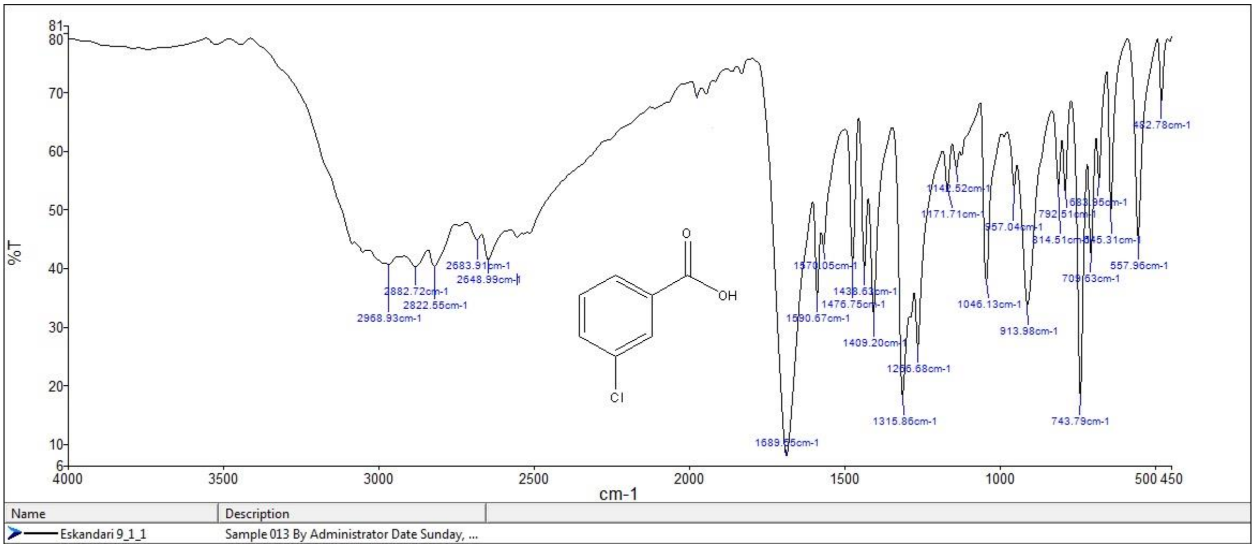

Fig. S15: FTIR spectrum of 3-Chlorobenzoic acid

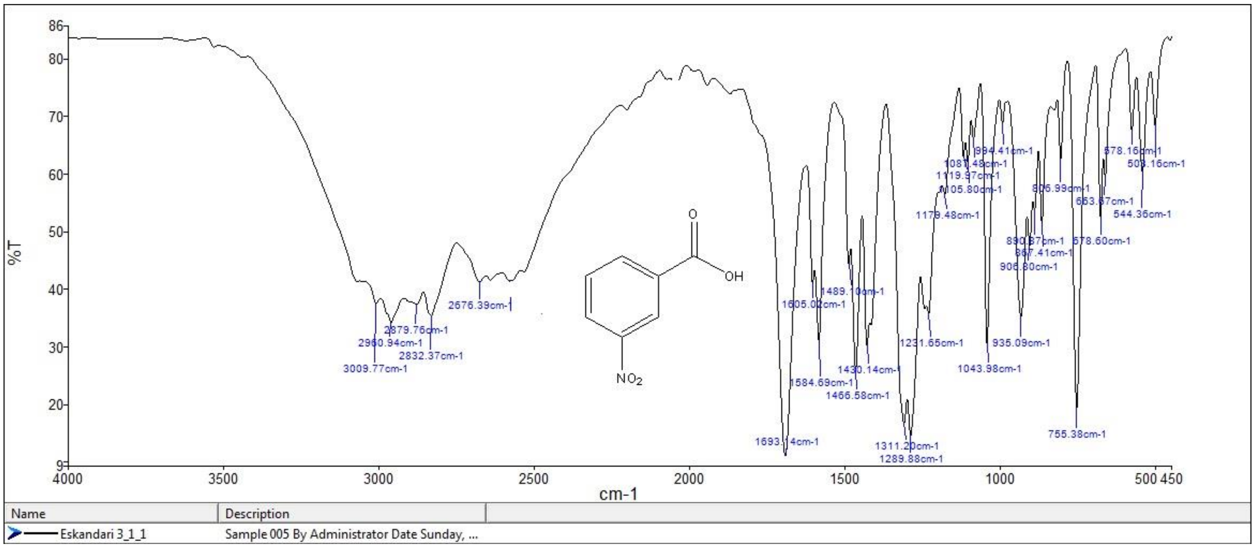

Fig. S15: FTIR spectrum of 3-Nitrobenzoic acid

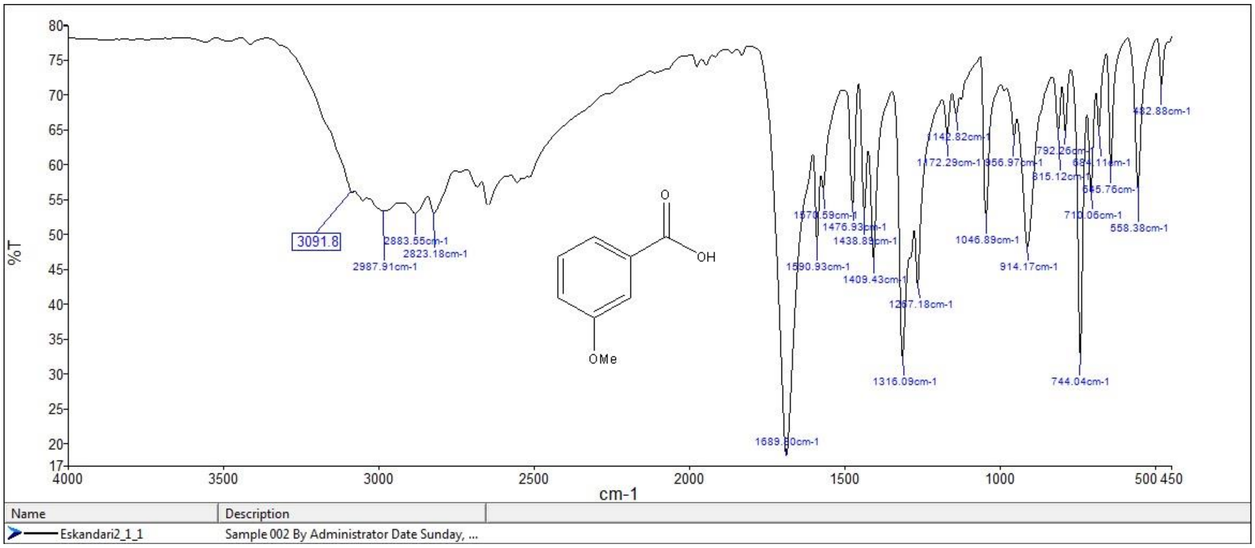

Fig. S16: FTIR spectrum of 3-Methoxybenzoic acid

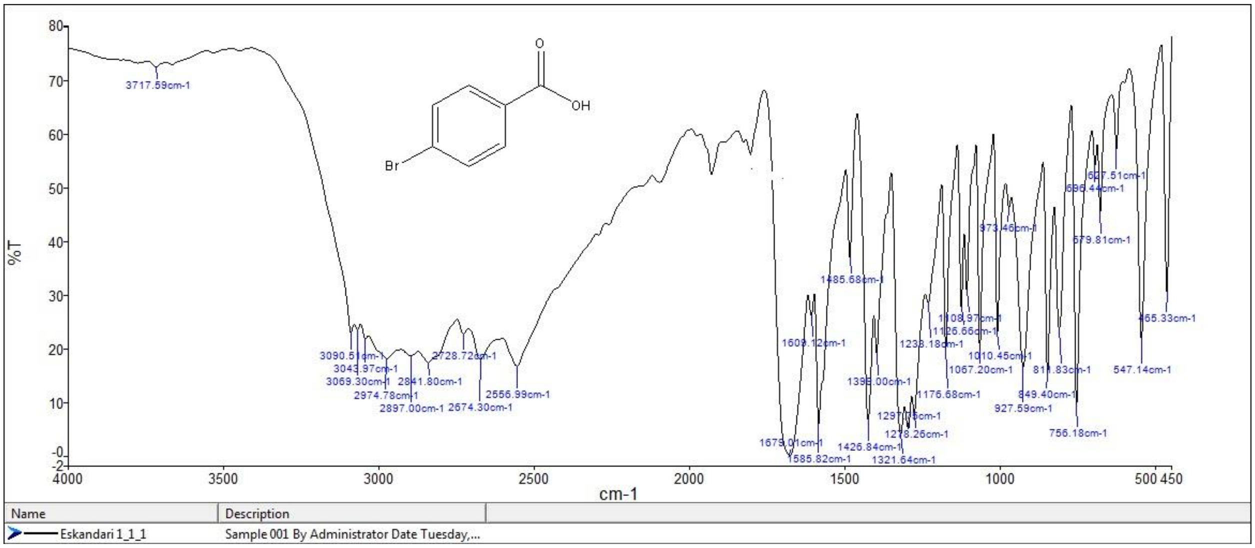

Fig. S17: FTIR spectrum of 4-Bromobenzoic acid

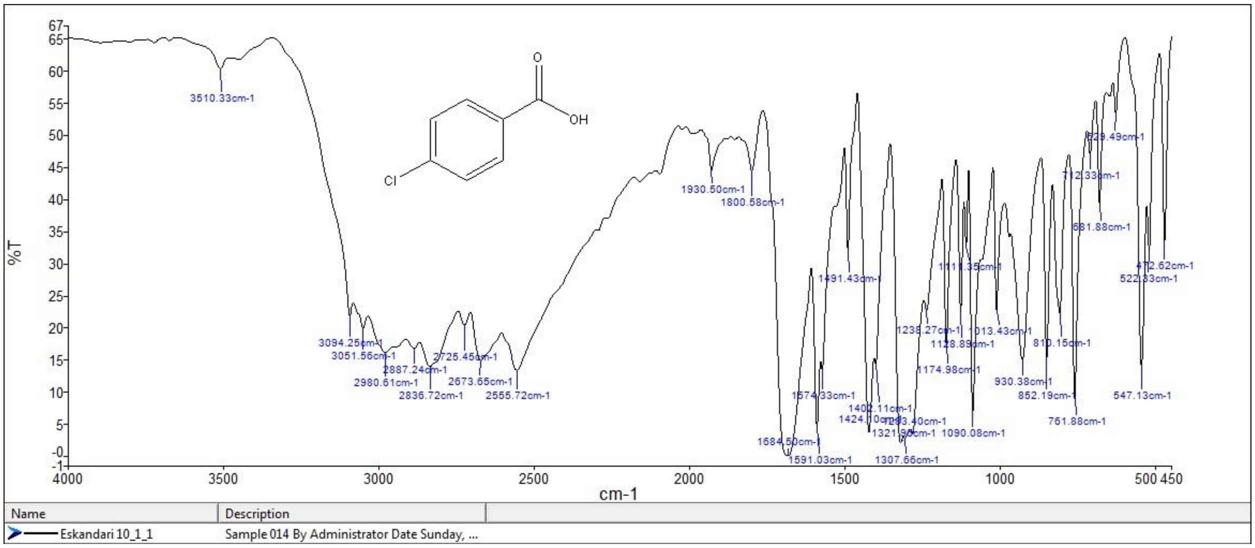

Fig. S18: FTIR spectrum of 4-Chlorobenzoic acid

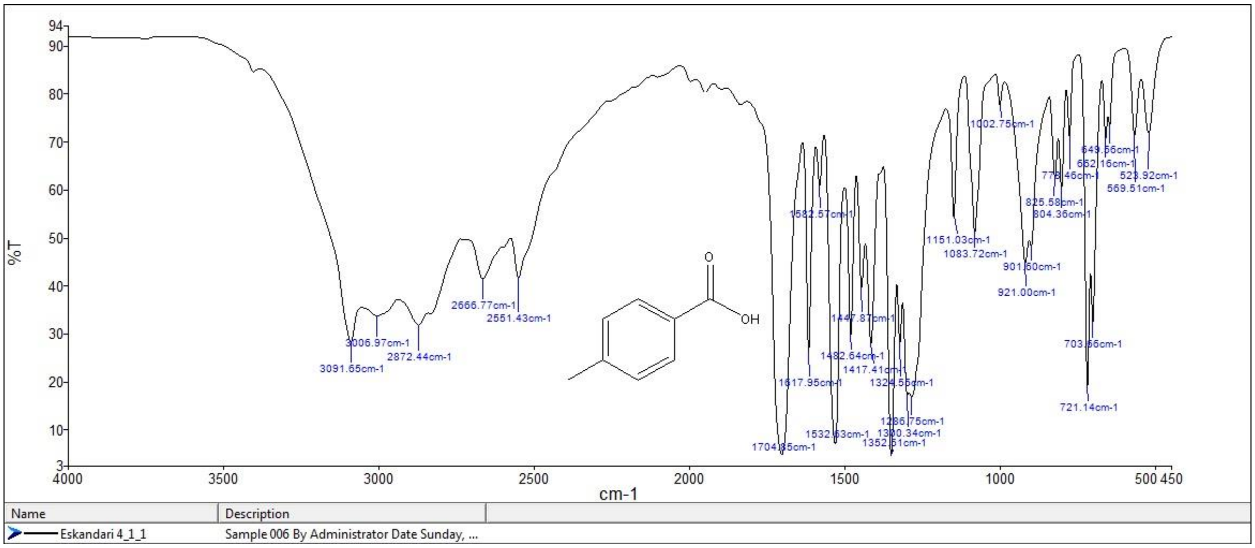

Fig. S19: FTIR spectrum of 4-Methylbenzoic acid

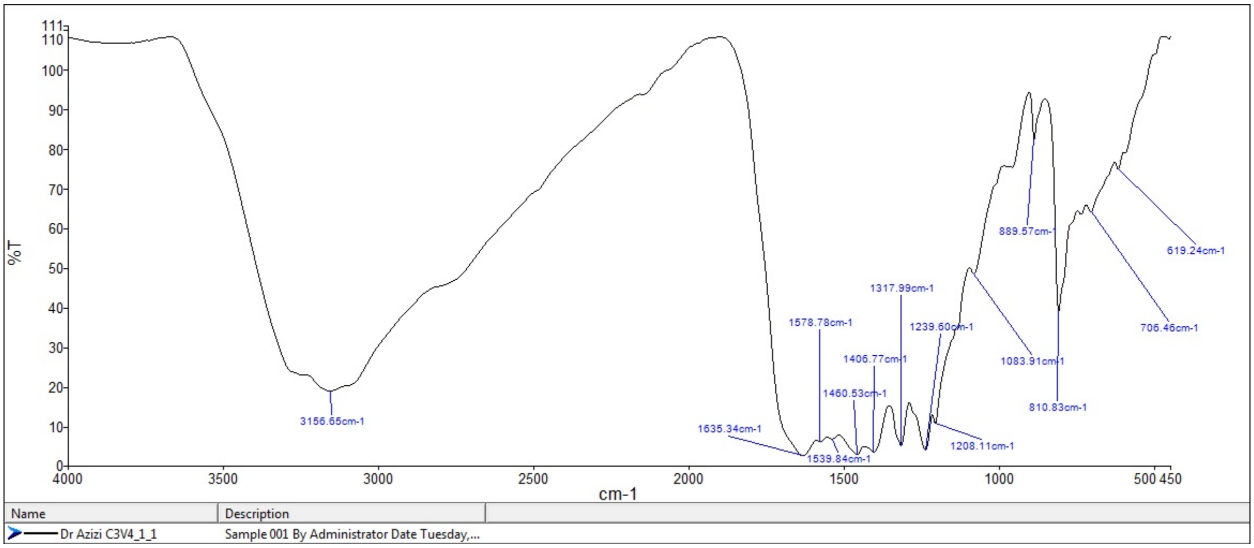

Fig. S20: FTIR spectra of Co(salen)@g-C_3N_4
